# Saikosaponin b1 Attenuates Liver Fibrosis by Blocking STAT3/Gli1 Interaction and Inducing Gli1 Degradation

**DOI:** 10.1002/EXP.70000

**Published:** 2025-02-03

**Authors:** Meiyu Shao, Xiaoqing Zhang, Jiamei Sun, Hongyan Dong, Xin Han, Qiao Yang, Roufen Chen, Liteng Shen, Lei Xu, Lu Wang, Bo Zhu, Dongxin Tang, Shuosheng Zhang, Keda Lu, Mengyun Peng, Gang Cao

**Affiliations:** ^1^ School of Pharmacy, The First Affiliated Hospital Zhejiang Chinese Medical University Hangzhou P. R. China; ^2^ Innovation Institute for Artificial Intelligence in Medicine Zhejiang University Hangzhou China; ^3^ Institute of Bioinformatics and Medical Engineering School of Electrical and Information Engineering Jiangsu University of Technology Changzhou P. R. China; ^4^ Microbiology and Genetics Department University of Salamanca Salamanca Spain; ^5^ Department of Science and Education The First Affiliated Hospital of Guizhou University of Chinese Medicine Guiyang China; ^6^ College of Chinese Materia Medica and Food Engineering Shanxi University of Chinese Medicine Jinzhong China; ^7^ The Third Affiliated Hospital of Zhejiang Chinese Medical University Hangzhou China

**Keywords:** liver fibrosis, Ssb1 binding site of STAT3, STAT3/Gli1 interaction

## Abstract

Saikosaponin b1 (Ssb1), a natural oleanane‐type triterpenoid saponin, exhibits antifibrosis activity by inhibiting the activation of hepatic stellate cells (HSCs), but the specific underlying molecular mechanisms are unknown. Here, it is found that Ssb1 could directly bind with the signal transducer and activator of transcription 3 (STAT3) and effectively inhibit the activation of HSCs. Proteomic techniques and molecular simulation revealed that Ssb1 is mainly bound to the S319 residues of STAT3 in the coiled‐coil domain. Further studies indicated that Ssb1 binding with STAT3 inhibited its transcriptional activity, and regulated glioma‐associated oncogene‐1 (Gli1) expression in the Hedgehog signaling pathway. Besides, Ssb1 binding blocked interaction between STAT3 and Gli1, which promoted degradation of Gli1 protein by suppressor of fused homolog (SUFU) and the ubiquitin‐proteasome system. The loss function of Gli1 led to decreased expression of Bcl2 and promoted the apoptosis of activated HSCs. Moreover, STAT3 ablation abolished the Ssb1‐mediated antifibrotic effects. These findings show that STAT3 plays a vital role in Ssb1 treatment of liver fibrosis, and Ssb1 as a STAT3 inhibitor might be a promising therapeutic candidate for the treatment of hepatic fibrosis.

## Graphical abstract

Saikosaponin b1 attenuates liver fibrosis by competitively disrupting the interaction between signal transducer and activator of transcription 3 (STAT3) and glioma‐associated oncogene‐1 (Gli1) in the activated hepatic stellate cells (HSCs). The blocked interaction of STAT3 and Gli1 promotes the degradation of Gli1, inhibits Gli1 transcriptional activity and regulates the antiapoptotic mediator's (Bcl2) expression, which induces the activation of Bax and triggers the intrinsic or mitochondrial pathway of apoptosis.

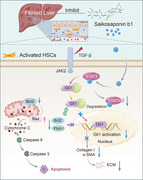

## 1 Introduction

Liver fibrosis is a pathological outcome of chronic liver injury‐induced wound healing and tissue regeneration resulting from viral, autoimmune, drug, cholestasis, and metabolic diseases [1]. Liver fibrosis is characterized by excess deposition of the extracellular matrix (ECM), and increased ECM is associated with tumorigenesis [2, 3]. Collagen types I and III and fibronectin are the main components of ECM, and they have been reported to promote precancerous cell growth via integrin signaling [3]. In addition, intensified ECM stiffness inhibits the differentiation of hepatocytes and disrupts the normal architecture of the liver, thus increasing the risk of hepatocellular carcinoma [4].

Activation of hepatic stellate cells (HSCs) is the major source of ECM and the key driver of liver fibrosis [5]. Quiescent HSCs are activated by profibrotic stimuli and transdifferentiated into activated HSCs (aHSCs), which can produce excess Collagen I and facilitate ECM formation [6]. HSCs can be activated by many factors, including soluble stimuli (reactive oxygen species, LPS, and apoptotic bodies) and paracrine stimuli (secreted by Kupffer cells, sinusoidal endothelium, and hepatocytes) [7]. Studies have shown that clearance of aHSCs from fibrotic liver through apoptosis or deactivation can promote fibrosis regression [8]. Thus, the elimination of aHSCs is an attractive strategy for clinical antifibrotic treatment [9].

Saikosaponin b1 (Ssb1) is one of the biologically active pentacyclic triterpenoid oleanolic acid derivatives from *Bupleurum chinense* DC, and it has been used for liver disease treatment throughout the long history of China [10, 11, 12]. Compared with the widely studied Ssa and Ssd, Ssb1 exerts less toxic side effects on normal hepatocytes, so it can alleviate liver diseases with no additional hepatoxicity [13]. Recent studies have elaborated the extensive pharmacological activities of Ssb1, including its anti‐inflammatory, antiviral, anticancer, and hepatoprotective effects [14, 15, 16]. Our previous study demonstrated that Ssb1 can effectively drive the apoptosis of aHSCs and the degradation of ECM in CCl_4_‐induced fibrotic models, therefore protecting mice against liver fibrosis [17]. However, the mechanism of Ssb1‐induced aHSCs apoptosis is poorly understood, which restricts the application of Ssb1 in liver fibrosis.

Based on our previous results, we explored the underlying mechanisms of Ssb1 that induce the apoptosis of aHSCs during liver fibrosis in this work. The results showed that Ssb1 could directly bind to the signal transducer and activator of transcription 3 (STAT3), an important positive transcription factor in tissue fibrosis, in aHSC cells [18]. The binding of Ssb1 with STAT3 inhibited the phosphorylation, dimerization, and nuclear translocation of STAT3, therefore downregulating its transcriptional activity [19]. RNA sequencing of STAT3‐knockdown (KD) HSCs revealed that the Ssb1 bound to STAT3 suppressed the activation of the Hedgehog (Hh) signaling pathway and decreased the expression of downstream antiapoptotic proteins. We also found that Ssb1 was effective in carbon tetrachloride (CCl_4_)‐ and thioacetamide‐induced fibrotic models, resulting in the regression of liver fibrosis through the apoptosis of aHSCs. This study indicates that Ssb1 might be a promising therapeutic candidate for the treatment of hepatic fibrosis.

## 2 Results

### 2.1 Ssb1 Inhibits Liver Fibrosis by Suppressing the Activation of HSCs

**FIGURE 1:**
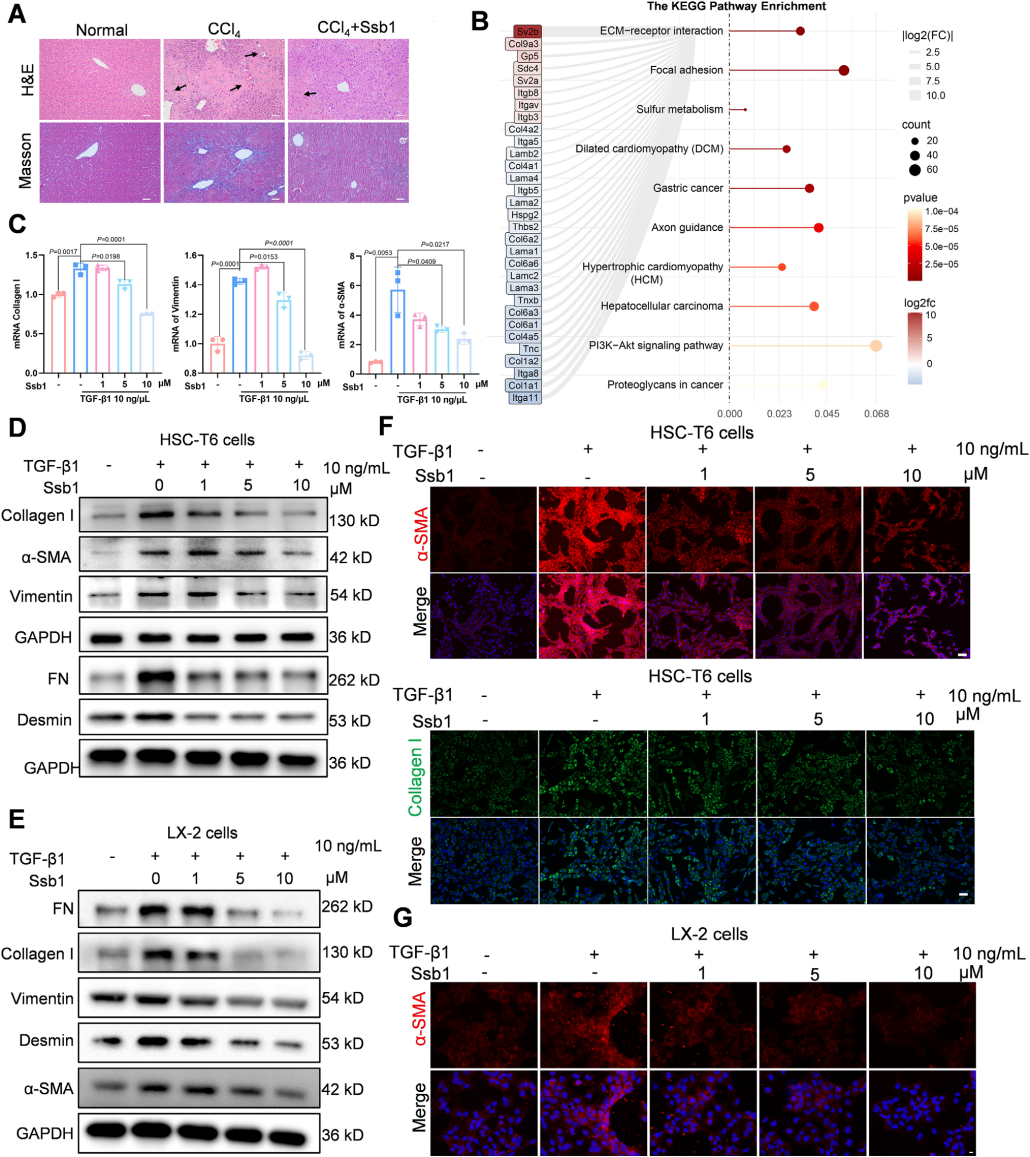
Ssb1 attenuated the activation of HSCs in vitro. (A) Representative H&E staining and Masson images for liver tissues (scale bars, 50 µm). (B) KEGG enrichment analysis of RNA‐sequencing data from differentially expressed genes between CCl_4_‐induced liver versus CCl_4_ treated with Ssb1. (C) mRNA levels of Collagen I, Vimentin, and α‐SMA after treating HSC‐T6 with different concentrations of Ssb1. (D,E) Protein levels of FN, Collagen I, Vimentin, Desmin, and α‐SMA detected by WB in (D) HSC‐T6 and (E) LX‐2 cells. (F,G) Immunofluorescence analysis of HSC‐T6 (scale bars, 50 µm) and LX‐2 cells (scale bars, 10 µm). All statistical data were presented as mean ± SD. Unpaired two‐tailed *t*‐test.

Our preliminary study found that Ssb1 could ameliorate CCl_4_‐induced hepatic fibrosis in mice by inducing the apoptosis of aHSCs, but the antifibrotic mechanism of Ssb1 still needs further exploration [17]. As shown in Figure 1A, Ssb1 administration decreased the deposition of collagen in the liver, indicating the antifibrosis capability of Ssb1. Liver tissues, including normal, CCl_4_ fibrotic group, and Ssb1‐treated fibrotic liver, were analyzed through transcriptome sequencing. Transcriptomic data revealed that differentially expressed genes were remarkably enriched in the ECM‐receptor interaction pathway after Ssb1 treatment, and many collagen genes were considerably decreased (Figure 1B). aHSCs are known to be the major producers of ECM components, which are key elements in liver fibrogenesis [20]. Therefore, we hypothesized that Ssb1 alters the function of aHSCs and decreases ECM decomposition.

Rat HSCs (HSC‐T6 cells) were used to examine the antifibrosis capability of Ssb1. After TGF‐β1 stimulation, elevated levels of fibrosis biomarkers (α‐smooth muscle actin [α‐SMA] and Collagen I proteins) were observed and remained for up to 24 h in the HSC‐T6 cells (Figure S1A). On the basis of the cell viability assay of Ssb1 at the 24 h time point (Figure S1B,C), hepatocytes that tolerated Ssb1 concentrations of 1, 5, and 10 µM were selected to assess the effects of Ssb1 on HSCs. The results indicated that the mRNA levels of the HSC activation biomarkers, α‐SMA, collagen I, and vimentin were considerably inhibited after Ssb1 incubation (Figure 1C). Western blot (WB) assay showed that Ssb1 inhibited fibronectin (FN), collagen I, vimentin, α‐SMA, and desmin expression in the HSC‐T6 cells in a dose‐dependent manner, and 5 µM Ssb1 could efficiently suppress HSC activation (Figure 1D; Figure S1D). Human HSCs (LX‐2 cells) were also used to verify the pharmaceutical effect of Ssb1 and similar results were obtained, as shown in Figure 1E and Figure S1E. Based on these findings, we performed immunofluorescence (IF) staining of HSC‐T6 (Figure 1F) and LX‐2 (Figure 1G) cells to visualize the activation and suppression of HSCs. The results showed that Ssb1 decreased the expression of TGF‐β1‐induced α‐SMA and Collagen I and suggested that Ssb1 could reduce TGF‐β1‐induced HSC activation and matrix protein expression.

### 2.2 Identification of STAT3 as the Direct Binding Protein of Ssb1

**FIGURE 2:**
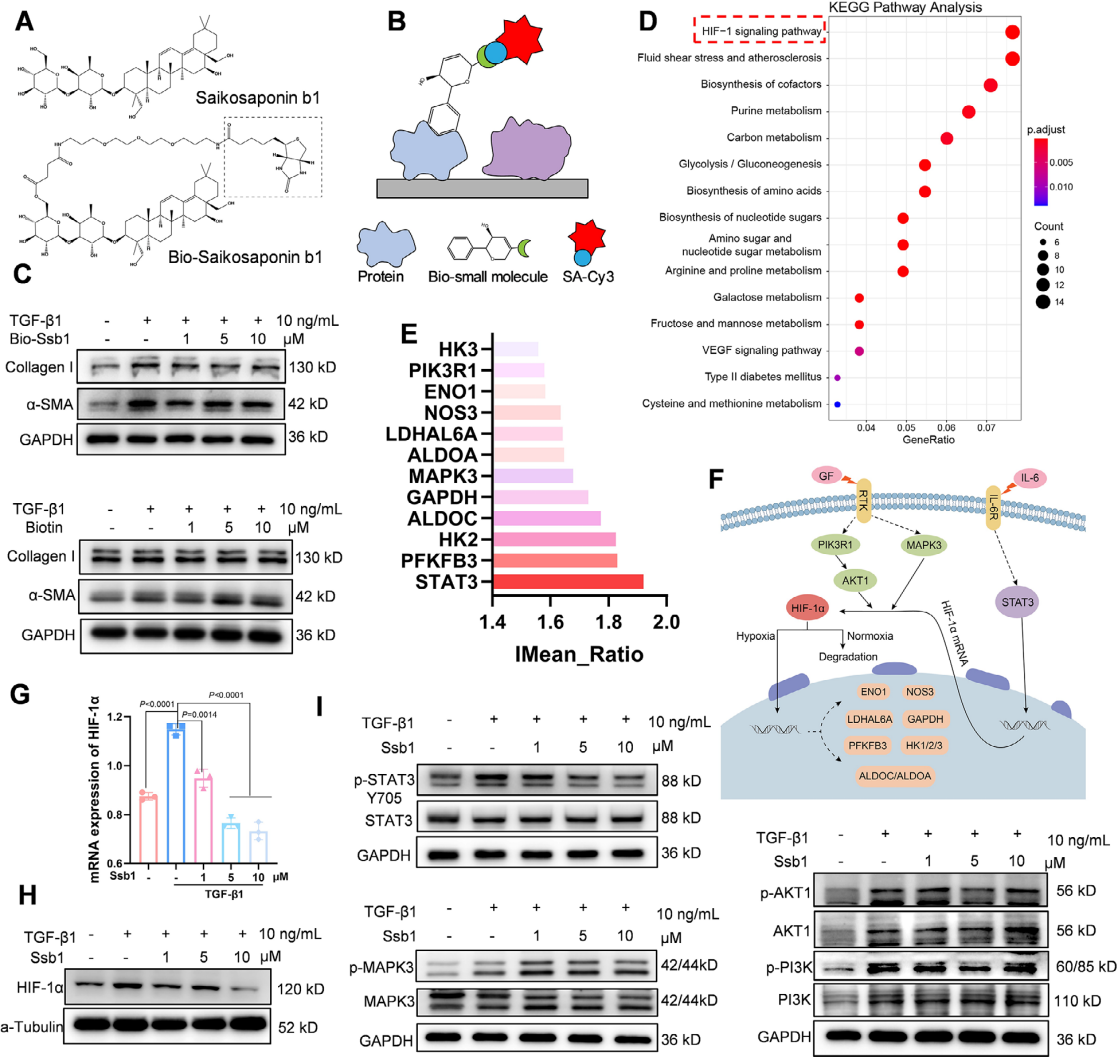
Identification of Ssb1‐binding proteins. (A) Chemical structure of Ssb1 and biotin‐labeled Ssb1 (Bio‐ssb1). (B) Schematic showing the identification of Ssb1‐binding proteins using microarrays fabricated with recombinant human proteins. (C) Bio‐Ssb1 retains protective activity against HSC activation. (D) KEGG enrichment analysis showing the binding partners of Ssb1. (E) IMean_Ratio of Ssb1 and a binding protein in the protein array. (F) Distribution of Ssb1‐binding proteins in the HIF‐1 signaling pathway. (G) mRNA and (H) protein levels of HIF‐1α after treating HSC‐T6 with different concentrations of Ssb1. (I) Protein level of p‐STAT3, p‐MAPK3, and p‐AKT1 after treating HSC‐T6 with different Ssb1 concentrations.

A biotin‐modified probe of Ssb1 was synthesized to explore the potential binding proteins for HSC inactivation and identify the antifibrosis mechanism of Ssb1. First, the biotin‐labelled Ssb1 (Bio‐Ssb1) binding of recombinant proteins to the HuProt human proteome microarray was assayed using Cy3‐conjugated streptavidin to screen direct‐binding proteins (Figure 2A,B). Given that Bio‐Ssb1 retained the inhibition activity of fibrotic protein expression in the activated HSC‐T6 cells, biotin modification did not influence the interaction between Ssb1 and proteins (Figure 2C and S2A). Second, Ssb1 positive binding proteins were further refined in accordance with the calculated signal‐to‐noise ratio (SNR) to examine the importance of Ssb1–protein interaction after background correction. Last, these proteins were analyzed through KEGG pathway enrichment. The results showed that Ssb1 binding proteins were preferably enriched in the HIF‐1 signaling pathway (Figure 2D), and 14 Ssb1 directly binding proteins were involved in this pathway. These proteins are listed with the IMean_Ratio in Figure 2E. IMean_Ratio was defined as the signal intensity ratio of the experimental group to the competition group pretreated with Ssb1. Among the proteins, STAT3 had the most remarkable binding and had the highest IMean_Ratio, indicating that Ssb1 was closely bound with STAT3.

In the HIF‐1 signaling pathway, we found that Ssb1 probably regulated HIF‐1α expression by directly binding to PI3K/AKT1, MAPK3, and STAT3 (Figure 2F). Ssb1 inhibition of HIF‐1α in the aHSCs was verified via quantitative polymerase chain reaction (qPCR) and WB (Figure 2G,H; Figure S2B). Then, the phosphorylation of STAT3, MAPK3, and PI3K/AKT1 (Thr34) in activated HSC‐T6 was determined after Ssb1 treatment (Figure 2I). Only the phosphorylation of STAT3 was considerably inhibited by Ssb1 (Figure S2C), indicating that the binding of Ssb1 with STAT3 effectively interfered with its function. Previous researchers have observed increased tyrosine 705 (Tyr 705) phosphorylation of STAT3 in liver fibrosis, which can promote HSC proliferation and activation and contribute to liver fibrogenesis [21, 22]. Therefore, we hypothesized that STAT3 might be the direct target of Ssb1 for the treatment of liver fibrosis.

### 2.3 Ssb1 Directly Bound to STAT3

**FIGURE 3:**
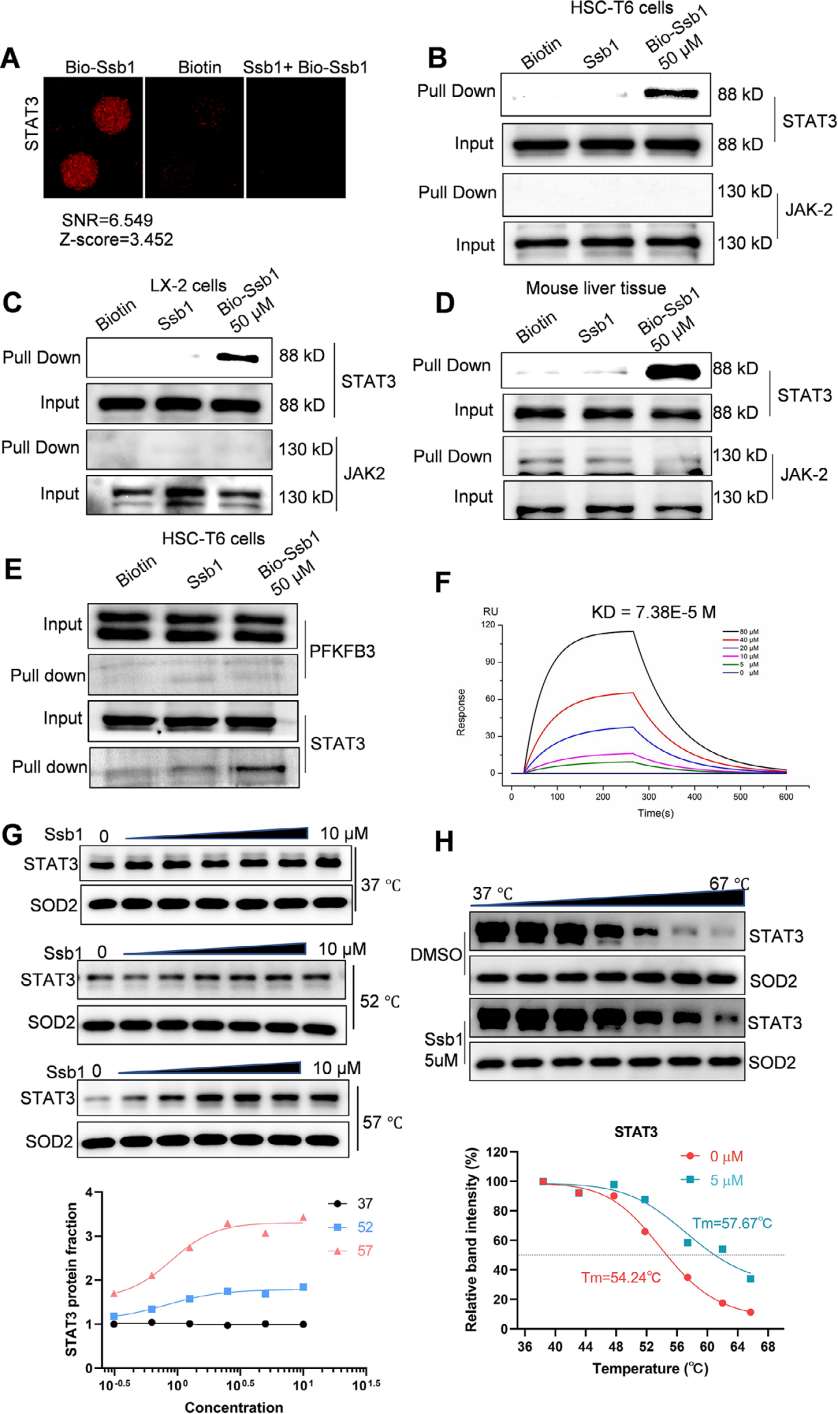
Ssb1 directly binds STAT3 protein. (A) Magnified image of Bio‐Ssb1 binding to STAT3 spot on the protein array. Signal‐to‐noise ratio (SNR) is shown. (B–D) Bio‐Ssb1 was added to streptavidin‐agarose beads and incubated. Biotin alone was used as a control. Lysates prepared from (B) HSC‐T6 cells, (C) LX‐2 cells and (D) mouse liver tissues were added to the streptavidin‐agarose beads with Bio‐Ssb1 using pull‐down assay. (E) Pull‐down assay of PFKFB3 by Bio‐Ssb1. (F) Surface plasmon resonance (SPR) analysis showing a direct interaction between Ssb1 and STAT3. (G) Dose‐dependent and (H) temperature‐dependent CETSA between Ssb1 and STAT3 in HSC‐T6.

Bio‐Ssb1–STAT3 binding in the protein microarray produced a robust SNR of 6.549, indicating that Bio‐Ssb1 could bind with STAT3 and that label‐free Ssb1 pretreatment effectively blocked Bio‐Ssb1 binding (Figure 3A and Figure S3A). A biotinylated protein interaction pull‐down analysis was also conducted to directly capture STAT3 in cell and tissue lysates (Figure S3B) [23]. Bio‐Ssb1 was added for incubation with lysates from HSC‐T6/LX‐2 cells and mouse liver tissues; afterward, streptavidin‐agarose beads were added to perform pull‐downs of STAT3 and upstream JAK2 and interleukin‐6 (IL‐6). As indicated in Figure 3B–D, Bio‐Ssb1 remarkably pulled down the STAT3 protein in the lysates from cells and tissues, but no obvious interactions between JAK2 and Ssb1 or IL‐6 and Ssb1 were observed (Figure S3C,E). Collectively, these results confirmed that Ssb1 directly binds to STAT3 but not to upstream mediators. Furthermore, as shown in Figure 2E, PFKFB3 has the second high affinity with Ssb1, Bio‐Ssb1 binding with PFKFB3 was performed to verify the priority of STAT3. Results showed that no obvious PFKFB3 was pulled down by Bio‐Ssb1, indicating that Ssb1 preferred to bind with STAT3 in HSCs (Figure 3E). To validate the interaction of Ssb1 with the STAT3 protein, we used surface plasmon resonance (SPR) to evaluate the direct interaction at the molecular level [24]. We observed that Ssb1 interacted with the recombinant human STAT3 (rhSTAT3) protein with an equilibrium dissociation constant of 73.8 µM (Figure 3F). Then, on the basis of the principle that small molecules may increase protein stability and melting temperature (*T*_m_) by forming a ligand‐protein complex [25, 26], cellular thermal shift assays were performed to confirm the effect of Ssb1 binding on STAT3 stability. The isothermal dose‐response curve in Figure. 3G indicated that different‐concentration treatments of Ssb1 (0–10 µM) increased the thermal stabilization of STAT3. Moreover, 5 µM Ssb1 stabilized STAT3 with a *T*_m_ shift of 3.43°C, suggesting the intracellular binding of Ssb1 and STAT3 (Figure 3H).

### 2.4 Binding Modes of Ssb1 to STAT3 Protein

**FIGURE 4:**
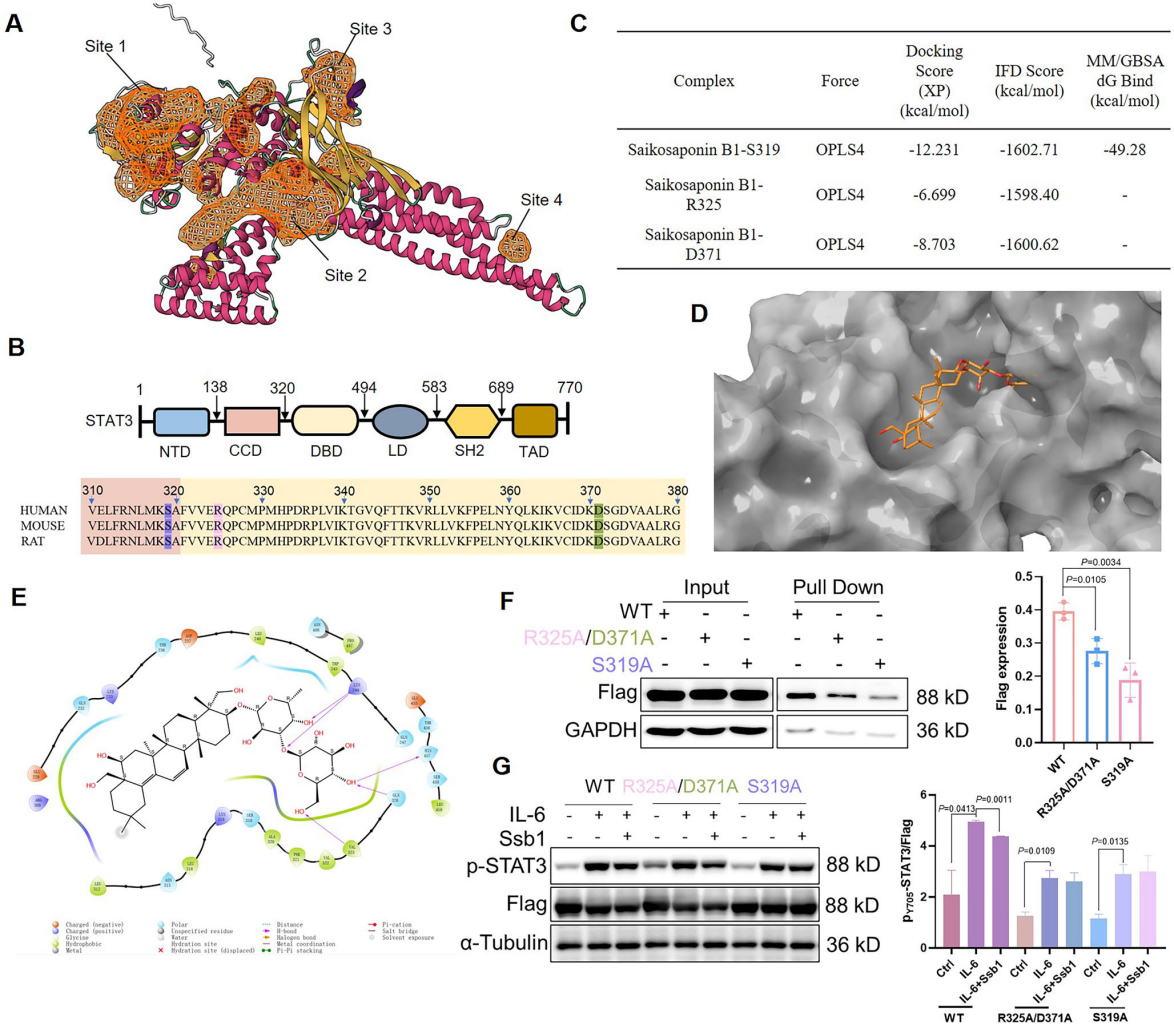
Mapping the binding sites of Ssb1 on STAT3. (A) DeepSite prediction of the center of each candidate pocket of STAT3 protein. (B) Diagrams of STAT3 domains. (C) Docking scores (XP and IFD) of the complexes of Ssb1 with distinct amino acid binding pockets of STAT3. (D) Overall conformation of the Ssb1 complex with STAT3‐S319 binding modes after molecular dynamics. (E) 2D diagram depicting the protein‐ligand interaction in the Ssb1 complex with STAT3‐S319. (F) 293T cells were transfected with wild‐type STAT3 or mutant STAT3. Lysates were added to pull‐down assays to detect Ssb1 binding using pull‐down assay. (G) S319A mutation blocked the inhibitory effect of Ssb1 on STAT3 phosphorylation. [Correction added on 30‐July‐2025 after first online publication: Figure 4 and the caption for Figure 4A have been updated.]

To identify the binding sites of STAT3 protein, the online deep‐learning‐based prediction tool, DeepSite was applied. The center of each candidate pocket of STAT3 protein was calculated together with a confidence score (Score), and four candidate centers were validated with scores ≥ 0.89: Site 1: Score = 0.9981, Center = (21.87, 11.39, −39.03); Site 2: Score = 0.9882, Center = (−0.13, −6.61, 4.97); Site 3: Score = 0.9764, Center = (−10.13, 31.39, −1.03); Site 4: Score = 0.8910, Center = (−40.13, −8.61, 46.97) (Figure 4A). The functional relevance of the predicted pockets was assessed by constructing a 20 Å radius sphere around the Deepsite center, and residues whose closest atom lay within 20 Å of the pocket center was considered to be in the vicinity of the site. Ser319 (chain A, 18.82 Å) as a peripheral modulatory residue and Arg325 (chain A, 16.97 Å) as a direct binding residue of ligand were identified around Site 2, Asp371 (chain A, 11.67 Å) was identified near Site 3, and no functionally relevant residues reported in the literature were found within the 20 Å neighborhood of Site 1 and Site 4. Evaluation of the common structural motifs of STAT3 revealed that the potential residues were mainly distributed in the coiled‐coil domain (CCD, residues 139–320), and the DNA‐binding domain (DBD, residues 321–494), as indicated in Figure 4B. Glide‐XP docking and induced fit docking (IFD) were applied to elucidate the binding mode of Ssb1 and STAT3. The results showed that the complex formed by Ssb1 with S319 had excellent XP docking and IFD scores, namely, −12.231/−1602.71 kcal·mol^−1^ (Figure 4C). Binding free energy was evaluated using the MM/GBSA method, which revealed that the binding free energy of the Ssb1/STAT3‐S319 complex was −49.28 kcal·mol^−1^. This result indicates strong binding (Figure 4C). To gain insights into the mode of action of Ssb1 in the STAT3‐S319 pocket, we analyzed the molecular dynamics of the last frame conformations of the Ssb1/STAT3‐S319 complex. Ssb1 could be effectively embedded within the STAT3‐S319 pocket and formed multiple hydrogen‐bonding interactions with the key residues, such as LYS244, HIS457, GLN326, and VAL323, suggesting that the binding of Ssb1 with S319 site of STAT3 showed higher affinity and stability compared with other pockets (Figure 4D,E; Figure S4A).

As S319 of STAT3 was calculated to be the prominent site for Ssb1 binding, we further mutated the three potential residues into Ala to confirm their involvement in the Ssb1‐STAT3 interaction. Two plasmids, namely, pcDNA3‐flag‐ratSTAT3‐R325A/D371A, pcDNA3‐flag‐ratSTAT3‐S319A, were constructed based on their distribution in the STAT3 domains. pcDNA3‐flag‐ratSTAT3‐WT was used as the control. These plasmids were transfected into human embryonic kidney 293 (HEK‐293T) cells to explore the interactions between Bio‐Ssb1 and mutated STAT3 via pull‐down assays. The results showed that wild‐type STAT3 (WT) was pulled down by Bio‐Ssb1. However, the S319A and R325A/D371A mutants, especially S319A, reduced the interaction of Bio‐Ssb1, and a decreased amount of STAT3 proteins were pulled down by Bio‐Ssb1 in 293T cell lysates (Figure 4F). Moreover, we further estimated the influence of the mutants on the inhibition of STAT3 phosphorylation by Ssb1. WT or STAT3 mutants transfected on 293T cells were stimulated with IL‐6 to active STAT3, and Ssb1 was added to examine the inhibition effect on STAT3 phosphorylation. The S319A or R325A/D371A variants abolished the Ssb1‐mediated inhibition of STAT3 phosphorylation (Figure 4G; Figure S4B). Collectively, these results demonstrate that STAT3‐S319 may be the prominent site for Ssb1 selectively binding, and the binding between Ssb1 and STAT3 further limited STAT3 phosphorylation.

### 2.5 STAT3 is Activated in Liver Fibrosis

**FIGURE 5:**
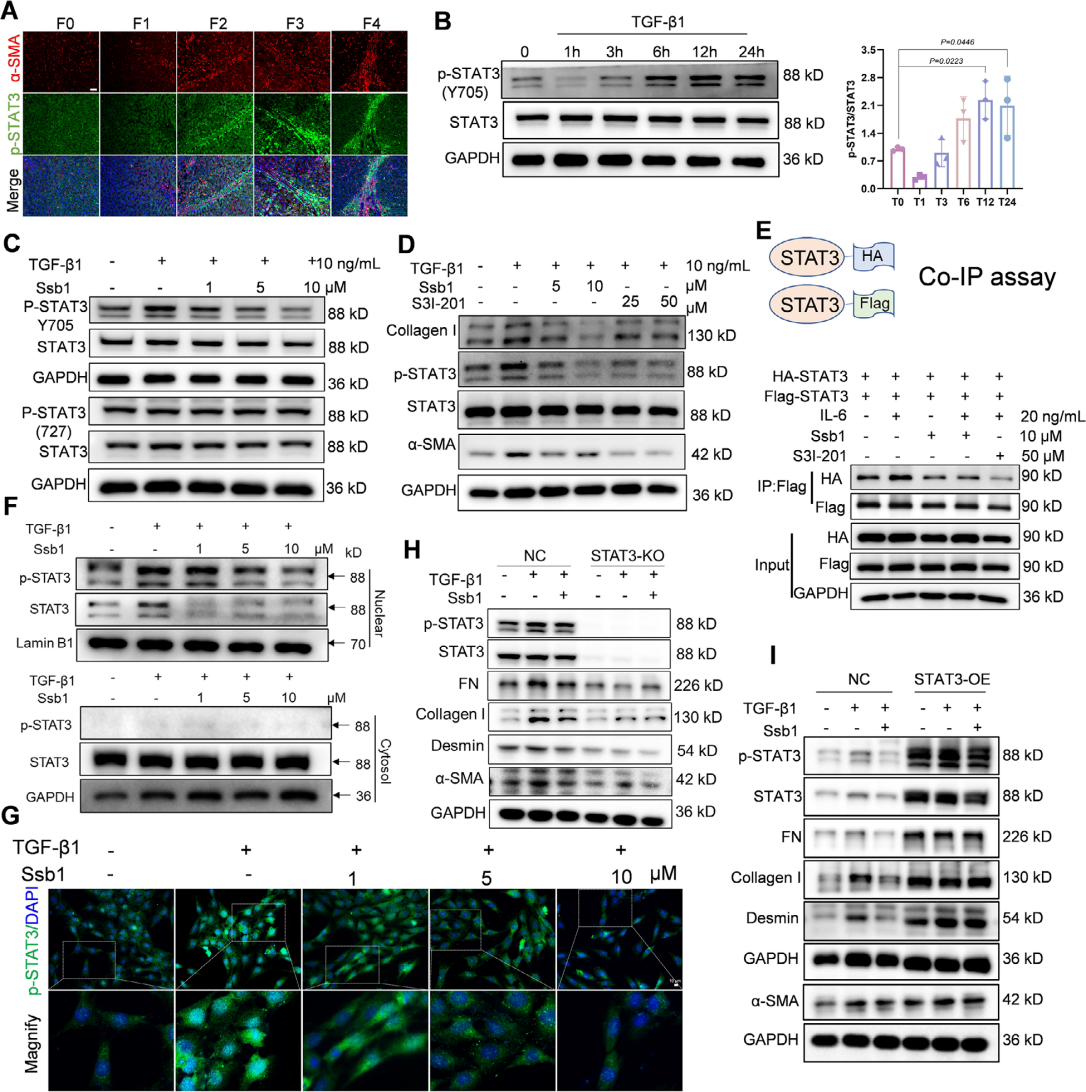
Ssb1 blocks STAT3 activation in activated HSCs. (A) Representative immunofluorescence of clinical human liver tissues with different stages of liver fibrosis. Scale bars, 50 µm. (B) Time course of p‐STAT3 induction in response to TGF‐β1 stimulation in HSC‐T6. (C) Protein levels of phosphorylated STAT3 (Try 705 and Ser 727) were detected by WB. (D) HSC‐T6 cells were treated with different concentrations of Ssb1 and S3I‐201 (STAT3 inhibitor) for 24 h. WB analyses of the expression of α‐SMA, Collagen I, FN, and Desmin. (E) Co‐IP assay indicates that Ssb1 disrupts the dimerization of STAT3 with or without IL‐6 stimulation. 293T cells were transfected with the indicated vectors and treated with IL‐6 (20 ng/mL) and Ssb1 for 24 h before harvest, followed by IP assay using a Flag antibody. (F) Ssb1 inhibits TGF‐β1‐induced STAT3 nuclear translocation. HSC‐T6 cells were treated with TGF‐β1 and Ssb1 for 24 h. Nuclear and cytosolic fractions were extracted and probed for p‐STAT3/STAT3. Lamin B1 and GAPDH were used as loading controls. (G) IF staining analysis of p‐STAT3 in HSC‐T6. (H,I) STAT3 were of significance to the antifibrotic effects of Ssb1. The protein levels of FN, Collagen I, Desmin, and a‐SMA following (H) STAT3 knockout or (I) STAT3 overexpression.

Pericarcinomatous tissue of patients with liver cancer was collected as fibrosis replacements to identify the pathological STAT3 activation at different stages of liver fibrosis [27]. The increased accumulation of collagen fibers and inflammation cells was positively correlated with liver fibrosis exacerbation (Figure S5A). The α‐SMA level and phosphorylated STAT3 were elevated and observed in coincident areas with the severity degree of fibrosis (F0–F4; Figure 5A). Similar results were obtained from the liver cirrhosis tissue and CCl_4_‐induced fibrotic liver of Balb/c mouse. STAT3 phosphorylation increased in the α‐SMA high‐expressed fibrosis area, and obvious nucleus translocation of p‐STAT3 was exhibited (Figure S5B–E). The STAT3 activation in the fibrotic liver was further quantified in bile‐duct‐ligation or CCl_4_‐induced mouse models, and considerably increased STAT3 Tyr 705 phosphorylation was observed (Figure S5F,G). Furthermore, the p‐STAT3 level of the Tyr 705 site was detected in the TGF‐β1‐stimulated HSC‐T6 cells at different time points. STAT3 phosphorylation increased at 6 h and reached the maximum level at 12 h; this level was maintained for up to 24 h, indicating that the activation of HSC‐T6 was accompanied by STAT3 Tyr 705 phosphorylation (Figure 5B).

### 2.6 Ssb1 Attenuates Liver Fibrosis by Blocking STAT3 Activation

A STAT3 inhibitor, S3I‐201, was used to determine if STAT3 activation blocking could inhibit aHSCs. Treatment with S3I‐201 effectively inhibited the phosphorylation of STAT3, reduced the differentiation of HSCs into myofibroblasts, and inhibited collagen synthesis with decreased Collagen I level (Figure S6A,B). STAT3‐knockout (STAT3‐KO) HSC‐T6 cells were established to exclude the STAT3 effect on HSC activation (Figure S6C,D). STAT3 KO decreased the mRNA and protein expression of HSC activation biomarkers and collagen synthesis. Conversely, overexpression of STAT3 (Figure S6E,F) increased the expression of fibrosis biomarkers and collagen synthesis. These data suggest that STAT3 activation in the liver HSCs was positively correlated with the development of liver fibrosis.

Given that Ssb1 could directly bind to STAT3, we further determined if Ssb1 inhibited TGF‐β1‐induced STAT3 activation and prevented HSC activation by targeting STAT3. As shown in Figure 5C and Figure S7A,B, Ssb1 binding inhibited STAT3 phosphorylation in Tyr 705 but did not influence Ser 727 phosphorylation in the HSC‐T6 and LX‐2 cells. Compared with S3I‐201, Ssb1 exhibited similar phosphorylation‐inhibition efficiency but at a lower concentration, indicating a potent STAT3 inhibition capability (Figure 5D and Figure S7C). STAT3 Tyr 705 phosphorylation has been reported to induce STAT3 dimerization and nuclear translocation and upregulate STAT3's transcriptional activity [28, 29]. STAT3 dimerization was subsequently monitored by STAT3 overexpression plasmids with different tags in 293T cells (Figure 5E). The coimmunoprecipitation (Co‐IP) assay revealed that two monomers of STAT3 could bind to each other after IL‐6 stimulation. Moreover, 10 µM Ssb1 binding disrupted STAT3 dimerization effectively even in the presence of IL‐6 and exerted an effect that was nearly equivalent to that of 50 µM S3I‐201 on inhibiting STAT3 dimerization induced by IL‐6 (Figure 5E and Figure S7D). STAT3 translocation into the nucleus was also measured (Figure 5F). The results showed that STAT3 mainly accumulated in the cytoplasm, p‐STAT3 was mainly translocated to the nucleus, and Ssb1 treatment reduced nuclear p‐STAT3 translocation in the HSC‐T6 cells (Figure S7E). p‐STAT3 nuclear translocation was further assessed using IF staining in HSC‐T6 and LX‐2 cells, and visually decreased p‐STAT3 translocation in the nucleus was observed after Ssb1 incubation. This observation suggests that direct binding of Ssb1 successfully blocked STAT3 Tyr 705 phosphorylation, dimerization, and nuclear translocation (Figure 5G and Figure S7F).

STAT3‐KO and STAT3‐KD HSC‐T6 cells were constructed and used to determine if the antifibrotic effects of Ssb1 are dependent on STAT3 (Figure 5H and Figure S8A,B). In the STAT3‐KD cells, the inhibition of HSC‐T6 cell activation by Ssb1 was partially abrogated (Figure S8C,D). When STAT3 expression was excluded, Tyr 705 phosphorylation and the TGF‐β1‐induced expression of fibrotic markers decreased. Ssb1 could not further inhibit the expression of Collagen I, α‐SMA, and other fibrotic markers (Figure S8E,F). Furthermore, when STAT3 was overexpressed in the HSC‐T6 cells, the fibrotic biomarkers were dramatically promoted at mRNA and protein levels (Figure 5I and Figure S8G,H). In the STAT3‐OE cells, Ssb1 treatment still inhibited STAT3 activation and reversed the STAT3‐mediated expression of the fibrotic markers. Collectively, these results suggest that Ssb1 inhibited STAT3 activation in the HSCs and attenuated liver fibrosis in a STAT3‐dependent manner.

### 2.7 Ssb1 Inhibits the Hh Signaling Pathway Through Regulating Expression of Gli1

**FIGURE 6:**
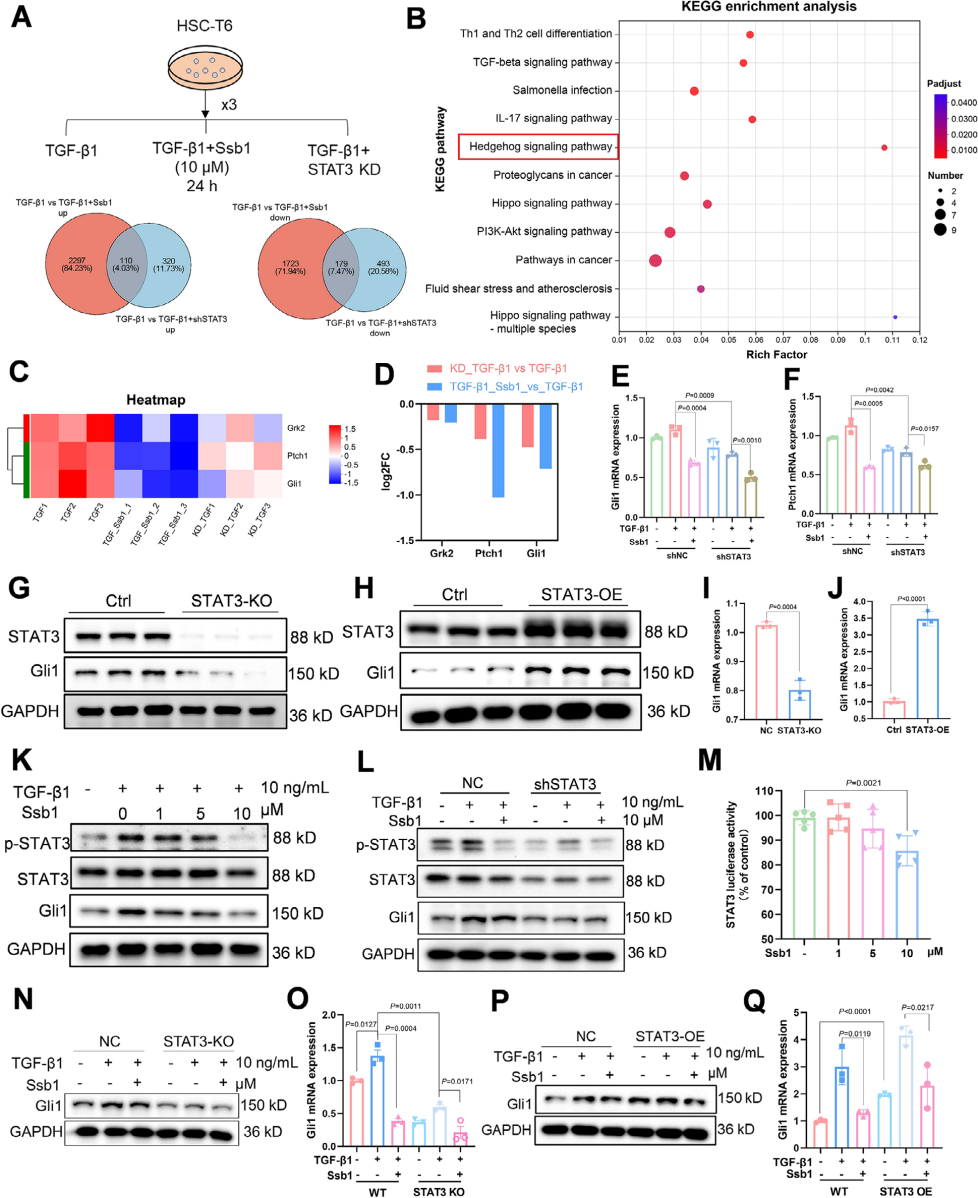
Ssb1 regulates the Hedgehog signaling pathway though targeting STAT3. Normal HSC‐T6 were treated with TGF‐β1 and SSb1, and STAT3‐knockdown HSC‐T6 were treated with TGF‐β1. RNA sequencing was carried out. (A) Venn diagram of upregulated (left) and downregulated (right) differentially expressed genes between TGF‐β1 versus TGF‐β1+SSb1 and TGF‐β1 versus shSTAT3+TGF‐β1. (B) KEGG enrichment of RNA‐sequencing data from differentially expressed genes. (C) Differentially expressed genes in the Hedgehog signal pathway, (D) and their log2 FC value. (E,F) mRNA levels of Gli1 and Ptch1 following STAT3 knockdown. (G–J) Protein and mRNA levels of Gli1 following STAT3 knockout or STAT3 overexpression. (K) Protein levels of Gli1 of HSC‐T6 treated with TGF‐β1 and different Ssb1 concentrations for 24 h. (L) Protein level of Gli1 in STAT3‐KD HSC‐T6. (M) STAT3 luciferase assay in 293T cells. 293T cells with a transfected Renilla luciferase vector and STAT3 luciferase reporter were stimulated with IL‐6 (20 ng/mL). Normalized luminescence was plotted as STAT3 luciferase activity (%). (N–Q) Protein and mRNA levels of Gli1 following treatment with TGF‐β1 and Ssb1 for 24 h in STAT3‐KO or STAT3‐overexpressed HSC‐T6.

RNA sequencing of HSC‐T6 cells was performed to investigate the molecular mechanisms of how Ssb1 inhibited the activation of HSCs by targeting STAT3 (Figure 6A). Differentially expressed genes (DEGs) regulated by Ssb1 and STAT3 were collected and analyzed with a Venn diagram. DEGs in “TGF‐β1 versus TGF‐β1 + Ssb1” group and “TGF‐β1 versus TGF‐β1 + shSTAT3” group were overlapped to recognize genes regulated by Ssb1 depending on STAT3. The results showed that 110 genes were upregulated and 179 genes were downregulated both in Ssb1‐treated aHSCs and TGF‐β1‐challenged STAT3‐KD HSC‐T6 cells (Figure 6A). KEGG functional annotation analysis of the DEGs revealed that they were enriched in the signal transduction category (Figure S9A). Next, the DEGs involved in signal transduction were screened and found to be enriched in the Hh signaling pathway with a high richness factor (Figure 6B). The heatmap of the DEGs in the Hh signaling pathway is displayed with their fold change in Figure 6C,D. We also conducted qPCR analysis to detect these genes’ expression levels in HSC‐T6 cells, and the results showed that Ssb1 treatment and STAT3 knockdown considerably promoted the downregulation of Gli1 and Ptch1 gene expression, a result that is consistent with the RNA sequencing result (Figure 6E,F). Gli1 is a downstream effector of the Hh signaling pathway [30]. Activated Gli1 translocates to the nucleus and activates the transcription of genes that promote cell survival and proliferation, such as Gli1, Ptch1, and Bcl‐2 [31]. Recent reports showed that STAT3 binds to the promoter of the Gli1 gene and controls Gli1 expression [32, 33, 34]. As presented in Figure 6G–J, knockout or overexpression of STAT3 in HSC‐T6 cells could regulate the expression of Gli1 at mRNA and protein levels, and Gli1 expression was dependent on STAT3 (Figure S9B).

Furthermore, the downregulation of Gli1 in the Ssb1‐treated HSCs was verified by WB. The results of the HSC‐T6 and LX‐2 cells showed that Ssb1 inhibited STAT3 phosphorylation and Gli1 expression in a dosage‐dependent manner (Figure 6K and Figure S9C–E). In STAT3‐KD HSC‐T6 cells, the Ssb1‐mediated inhibition of Gli1 expression became invalid (Figure 6L and Figure S9F). The STAT3 luciferase reporter plasmid was transfected into 293T cells and incubated with Ssb1 to determine if Ssb1 regulated Gli1 by binding with STAT3. The luciferase activity of STAT3 was considerably reduced by 10 µM Ssb1, which meant that the direct binding of Ssb1 with STAT3 downregulated its transcriptional activity, thus regulating Gli1 expression and blocking the Hh signaling pathway (Figure 6M).

Ssb1 regulation of Gli1 was also examined in STAT3‐KO and STAT3‐OE HSC‐T6 cells (Figure 6N–Q). Ssb1 treatment further downregulated Gli1 mRNA level, but no notable decrease of Gli1 protein expression was observed in the Ssb1 treated STAT3‐KO cells. By contrast, Ssb1 retained its inhibition compatibility and effectively decreased the level of Gli1 in the STAT3‐OE cells (Figure S9G,H). These results indicated that STAT3 was the dominant target protein of Ssb1 in the HSCs.

### 2.8 Ssb1 Blocks STAT3/Gli1 Interaction and Promotes Gli1 Degradation

**FIGURE 7:**
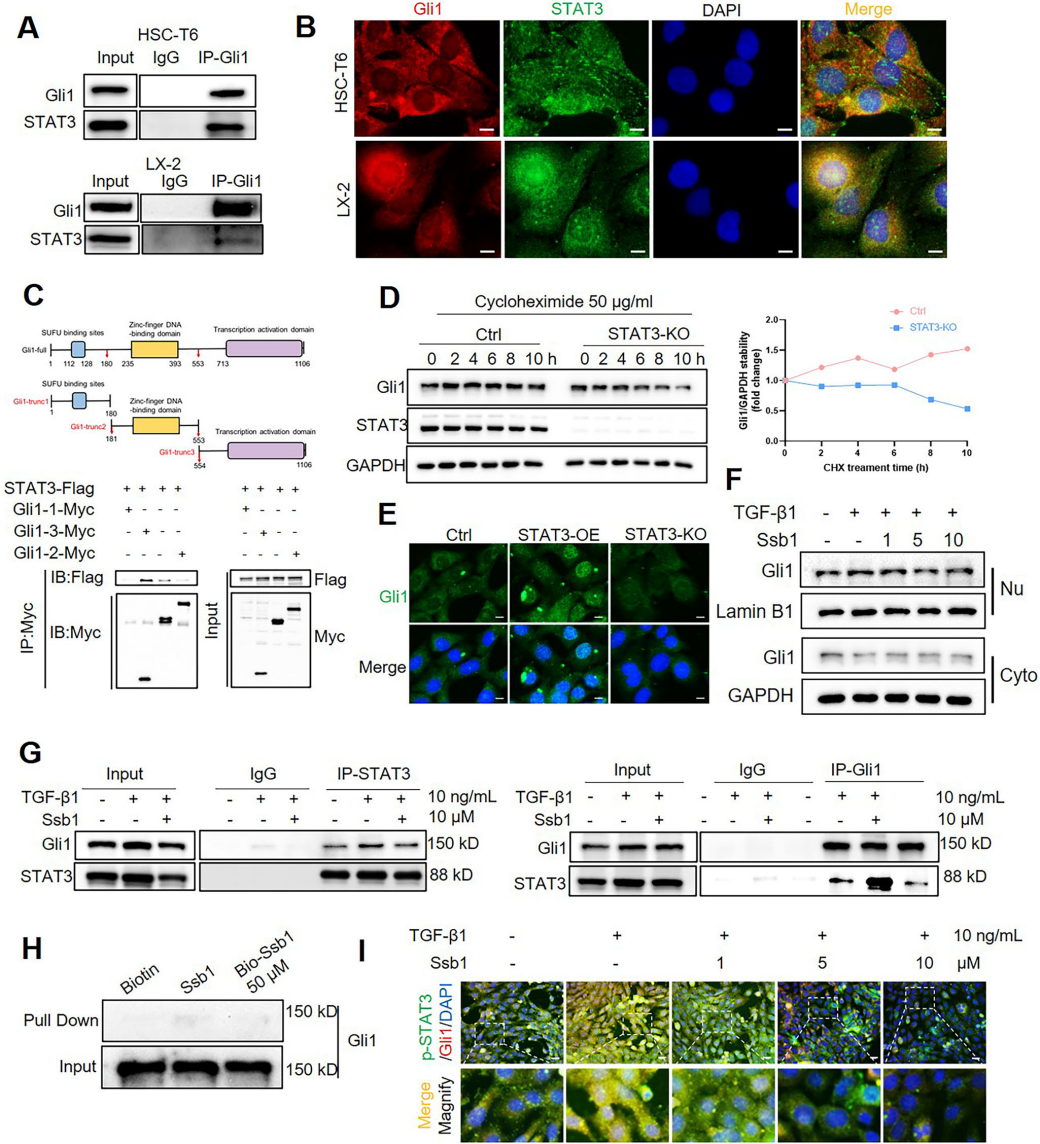
STAT3 forms a complex with Gli1 through the region of SUFU binding sites**. (**A) HSC‐T6 and LX‐2 cells were lysed and subjected to IP‐WB assay to determine the interaction between endogenous STAT3 and Gli1. (B) Immunofluorescence analysis of STAT3 and Gli1 distribution in HSC cell lines. DAPI was used to stain nuclei. Scale bars, 10 µm. (C) HEK‐293T cells were transiently transfected with STAT3‐Flag, Gli1‐trunc1‐Myc (SUFU binding sites), GLI1‐trunc2‐Myc (zinc‐finger DNA‐binding domain), and GLI1‐trunc3‐Myc (transcription activation domain). Cells were harvested 48 h after transfection followed by IP‐WB assay. (D) HSC‐T6 cells were treated with cycloheximide (CHX, 50 µg/mL) for the indicated times, and cell lysates were analyzed with WB with the indicated antibody. (E) Immunofluorescence analysis of Gli1 distribution in HSC‐T6 of STAT3 knockout or overexpression. Scale bar: 10 µm. (F) WB analysis of GLI1 distribution after Ssb1 treatment followed by nuclear cytosolic protein isolation in HSC‐T6. GAPDH and Lamin B1 were used as cytoplasmic and nuclei markers, respectively. (G) Ssb1 inhibits TGF‐β1‐induced STAT3‐Gli1 interaction. HSC‐T6 were treated with TGF‐β1 and Ssb1 for 24 h. Gli1‐STAT3 interactions were analyzed by coimmunoprecipitation. (H) Pull‐down assay of Ssb1 and Gli1 in HSC‐T6. (I) Expression and intracellular localization of Gli1 and pSTAT3 in HSC‐T6 with TGF‐β1 and Ssb1 treatment. Scale bar: 20 µm. [Correction added on 30‐July‐2025 after first online publication: In Figure 7D, “STAT3‐OE” is changed to “STAT3‐KO”.]

Aside from the direct regulation of Gli1 expression, recent evidence indicates that activated STAT3 can physically interact with Gli1 and lead to Gli1 enrichment in the promoters of target genes controlled by Gli1‐binding sites [32, 35]. In this study, IF staining of different‐state fibrotic clinical samples (F0–F4) revealed that the high expression of Gli1 was positively colocalized with STAT3 (Figure S10A). The Co‐IP assay of HSC‐T6 and LX‐2 cells was used to determine the interaction between endogenous Gli1 and STAT3 (Figure 7A). IF double staining results showed that both Gli1 and STAT3 were co‐localized in the nucleus (Figure 7B), which suggested a functional correlation between the two proteins. Gli1 has three main functional domains including SUFU binding sites, DNA binding domain, and C‐terminal transcriptional activation domain [36].

To identify the specific domain of Gli1 necessary for its interaction with STAT3, plasmids that express Myc‐tagged truncated Gli1 fragments of different functional domains were co‐transfected with Flag‐tagged STAT3 into HEK‐293T. Results showed that the truncated fragment containing SUFU binding sites has the most potent binding force with STAT3 (Figure 7C). It was concluded that STAT3 mainly formed a complex with the SUFU binding sites of Gli1. The function of SUFU is to sequester Gli1 in the cytoplasm and promote the degradation of Gli1 through the ubiquitin‐proteasome system [37]. Gli1 stability assay was performed by blocking endogenous protein synthesis with cycloheximide (CHX). Approximately one‐half of the endogenous Gli1 was degraded within 10 h in STAT3‐KO cell lines (Figure 7D). IF results showed that Gli1 was decreased in STAT3‐KO cells, while was highly concentrated within the nucleus in STAT3‐OE HSCs (Figure 7E). Gli1 abundance increased in the nucleus after TGF‐β1 stimulation (Figure S10B). WB results showed that Gli1 abundance decreased in the nucleus with the high dose of Ssb1 treatment (Figure 7F and Figure S10C).

The Co‐IP assay demonstrated that the STAT3/Gli1 interaction increased in the TGF‐β1‐activated HSC‐T6 cells, and Ssb1 administration effectively inhibited the protein–protein binding between STAT3 and Gli1 (Figure 7G). As demonstrated in this study, Ssb1 could directly bind with the STAT3 protein. The binding of Ssb1 with Gli1 was further examined. The pull‐down assay showed that Ssb1 did not interact with Gli1 in the HSC‐T6 and LX‐2 cells (Figure 7H and Figure S10D). Furthermore, pSTAT3/Gli1 colocalization in the HSCs was assayed by IF double staining. The results revealed that Ssb1 treatment inhibited the nuclear translocation of pSTAT3 and Gli1 in TGF‐β1‐induced HSC‐T6 and decreased their intranuclear colocalization (Figure 7I and Figure S10E).

### 2.9 STAT3/Gli1 Interaction Promotes Stability of Gli1 and Prevents aHSCs Apoptosis

**FIGURE 8:**
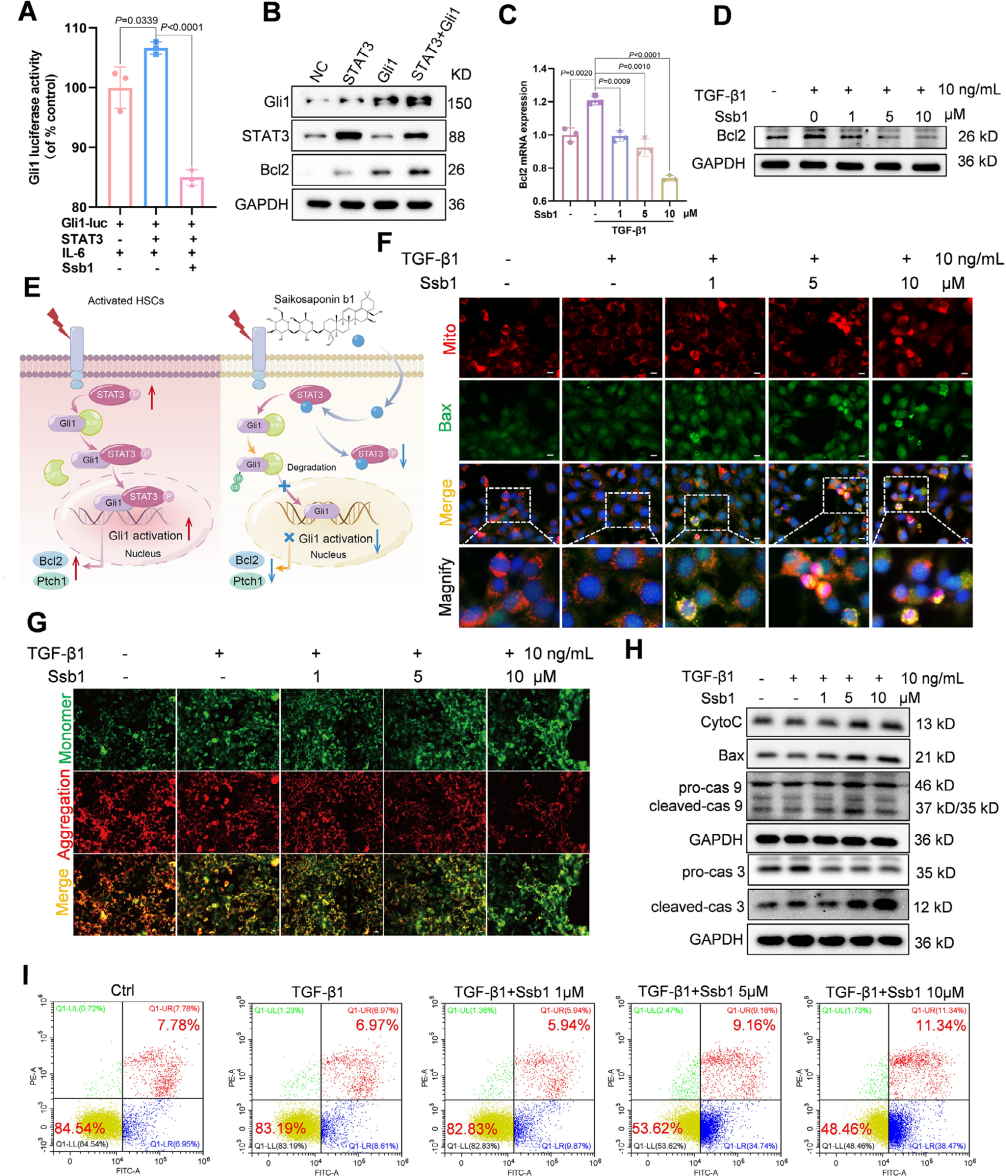
Ssb1 induces aHSCs apoptosis though targeting STAT3/Gli1. (A) Gli1 luciferase assay in 293T cells. Co‐overexpression of STAT3 in 293T cells with a transfected Renilla luciferase vector and Gli1 luciferase reporter were stimulated with IL‐6 (20 ng/mL) and Ssb1. (B) The expression of Bcl2 by co‐overexpression of STAT3‐Gli1. (C,D) The mRNA and protein levels of Bcl2 after treatment of HSC‐T6 cells with different Ssb1 concentrations. (E) The proposed mechanisms of Ssb1 in regulating STAT3/Gli1 interaction in aHSCs. (F) Immunofluorescence analysis of Bax distribution in HSC‐T6 after TGF‐β1 stimulated. Scale bar: 10 µm. (G) JC‐1 determined the mitochondrial membrane potential change after being treated with TGF‐β1 and different concentrations of Ssb1 for 24 h. Scale bar: 10 µm. (H) The protein expression levels of CytoC, Bax, cleaved‐Caspase‐3/9. (I) Apoptotic cells were detected by flow cytometry in Ssb1 treatment group.

Dual‐luciferase reporter assays of Gli1 showed that co‐overexpression of STAT3 markedly enhanced the transcriptional activity of Gli1, which was then considerably inhibited by Ssb1 incubation (Figure 8A). Therefore, we posit that Ssb1 may competitively inhibit the interaction between STAT3 and Gli1, promote the degradation of Gli1 through the ubiquitin‐proteasome system and decrease Gli1 nuclear import and transcriptional activity, resulting in downregulation of target genes and promoting the apoptosis of activated HSCs.

As a downstream target gene of Gli1 in the Hh signaling pathway, Bcl2 plays an important role in anti‐apoptosis and protects mitochondria from dysfunction [38]. In the STAT3‐KO and STAT3‐OE HSC‐T6 cells, expression of Bcl2 was positively correlated with the STAT3 level (Figure S11A,C). The mRNA levels of Bcl2 and Ptch1 were also detected, and a similar tendency was observed (Figure S11B,D). Further, Gli1 overexpression along increased the expression of Bcl2 without influence the expression of STAT3 (Figure S11E). Co‐overexpression of Gli1 with STAT3 markedly enhanced the expression of Bcl2 (Figure 8B; Figure S11F). Together, these observations confirmed that the STAT3‐Gli1 axis positively regulated Bcl2 expression. Specifically, the Hh signaling pathway could be regulated by STAT3 through Gli1.

Ssb1 treatment was further added to investigate its proapoptotic effect, and the expression level of Bcl2 was detected in HSC‐T6 and LX‐2 cells (Figure 8C,D; Figure S11G–I). The results showed that the expression of Bcl2 markedly changed with Ssb1 treatment. STAT3 knockout abolished the Ssb1 regulation ability of the Hh pathway (Figure S11J–L). After reinforcement of STAT3 expression, Ssb1 inhibition of Ptch1 and Bcl2 mRNA remained statistically significant, but no statistical difference was found at the protein level (Figure S11M–O). These results reveal that Ssb1 competitively disrupted the interaction between STAT3 and Gli1, which in turn promoted the degradation of Gli1, inhibited Gli1 nuclear translocation and transcriptional activity and regulated the antiapoptotic mediator's (Bcl2) expression in the activated HSCs (Figure 8E).

The apoptosis of aHSCs induced by Ssb1 was further assessed. Notably, a decrease in Bcl2 can activate mitochondria apoptotic pathways. The pro‐apoptotic activity of Bax and Bax‐induced cytochrome c release are inhibited by Bcl2, which retranslocates Bax from mitochondria to the cytosol, therefore preventing the accumulation of Bax at the mitochondrial outer membrane [39, 40]. Immunofluorescence staining (Figure 8F) was performed to prove that Bax was translocated to mitochondria after Ssb1 treatment. The mitochondrial membrane potential (MMP) was examined by JC‐1 staining [41], and HSC‐T6 cells were treated with different concentrations of Ssb1. The fluorescence of aggregated JC‐1 (red) decreased and the fluorescence of JC‐1 monomer (green) increased gradually with higher Ssb1 concentration, indicating the MMP loss and mitochondrial dysfunction induced by Ssb1 treatment (Figure 8G). As displayed in Figure 8H, the downregulation of Bcl‐2 by Ssb1 promoted the expression of Bax and the release of cytochrome c (Figure S12C). Furthermore, apoptotic enzymes involved in mitochondria‐controlled apoptosis, such as caspase 3, caspase 6 and caspase 9, were detected [42]. Treatment with Ssb1 remarkably increased the cleavage of caspase 3/6/9 proteins, therefore inducing the apoptosis of activated HSCs and attenuating the liver fibrosis process (Figure 8H; Figure S12A,B). Flow cytometry revealed that the proportion of apoptotic cells was increased by Ssb1 treatment in activated HSC‐T6 (Figure 8I). TUNEL assay is one of the most standardized methods to detect cell death by identifying DNA damage. Compared with the TGF‐β1 group, TUNEL‐positive cells increased significantly after Ssb1 treatment (Figure S12D). Therefore, the protein level of Bcl‐2 was downregulated with Ssb1 treatment, which induced the activation of Bax that results in MOM permeabilization (MOMP) and MOMP enables the release of cytochrome c released from the mitochondria into the cytoplasm which triggers the intrinsic or mitochondrial pathway of apoptosis.

### 2.10 Ssb1 Protects Mice From Liver Fibrosis Through STAT3 Inhibition

**FIGURE 9:**
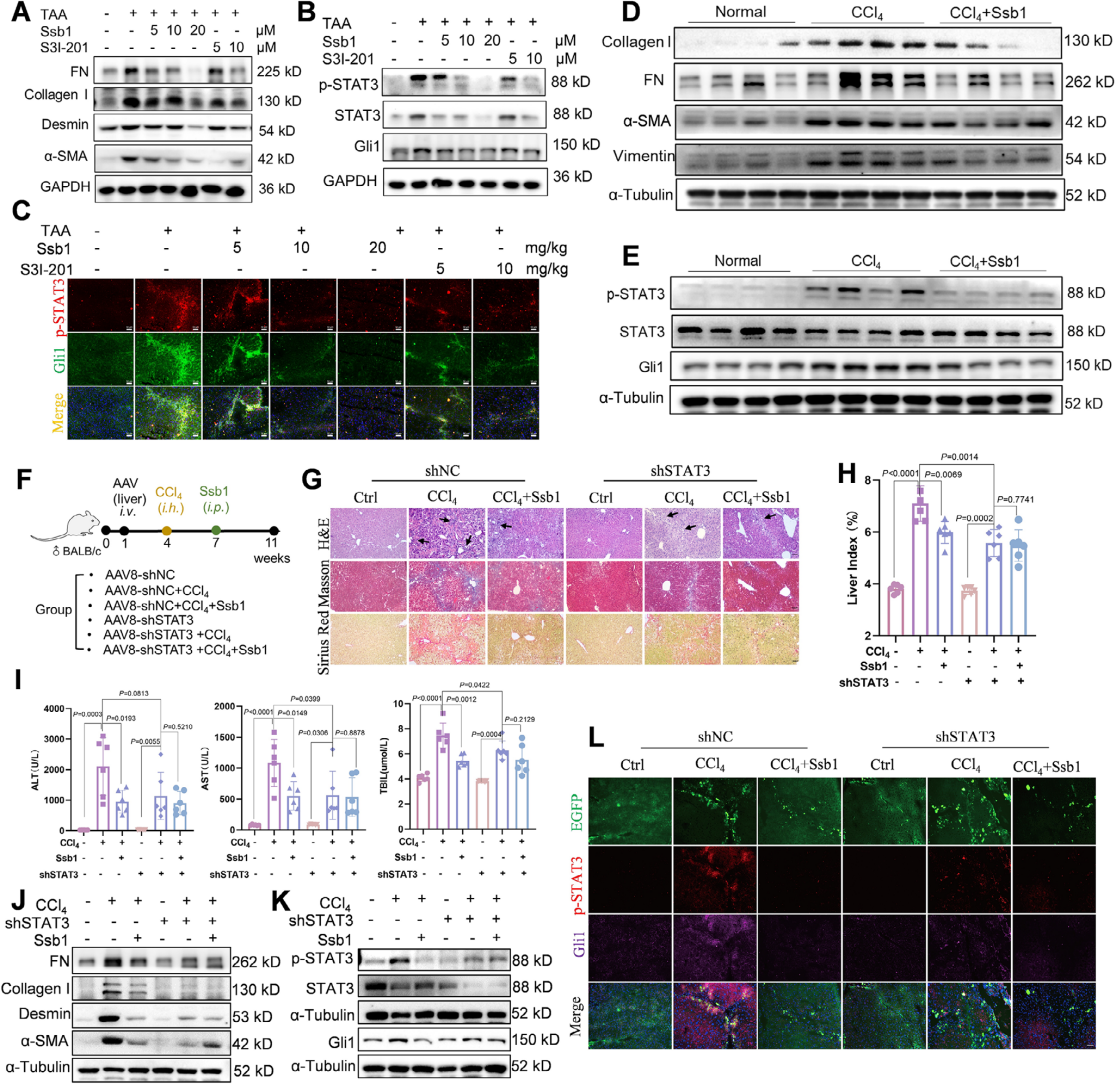
Effect of Ssb1 on liver fibrosis depends on STAT3. (A) Representative WB analysis of FN, Collagen I, Desmin, and α‐SMA in TAA‐induced mouse liver tissues treated with Ssb1 and S3I‐201. GAPDH was used as loading control. (B) Representative WB analysis of STAT3, p‐STAT3, and Gli1 in TAA mouse liver treated with Ssb1 and S3I‐201. (C) Immunofluorescence staining with p‐STAT3 and Gli1 of TAA‐induced fibrotic liver (scale bar, 50 µm). (D) Representative WB analysis of FN, Collagen I, Vimentin, and α‐SMA in CCl_4_‐induced liver tissues. α‐Tubulin was used as loading control. (E) Representative WB analysis of STAT3, p‐STAT3, and Gli1 in CCl_4_‐ induced liver tissues. (F) Scheme of the experimental approach. STAT3‐KD mice were treated with Ssb1 at the indicated doses for 4 weeks after being injected with CCl_4_ for 3 weeks. (G) Representative liver cross‐sections stained with H&E (scale bar, 50 µm), Masson's trichrome (scale bar, 50 µm), and Sirius Red (scale bar, 50 µm). (H) Liver index statistics of mice in each group. Each group contained six mice. (I) Concentrations of ALT, AST, and TBIL in mouse serum. (J) Representative WB analysis of FN, Collagen I, Vimentin, and α‐SMA in liver tissues. α‐Tubulin was used as loading control. (K) Representative WB analysis of STAT3, p‐STAT3, and Gli1 in liver tissues. (L) Immunofluorescence staining with p‐STAT3 and Gli1 (scale bar, 50 µm). All data were presented as mean ± SD. Unpaired two‐tailed *t*‐test.

To further verify the therapeutic effect of Ssb1 on liver fibrosis by STAT3 inhibition, we utilized a thioacetamide (TAA)‐induced liver fibrosis model to estimate the antifibrosis effect of Ssb1. S3I‐201 was adopted as a positive control to inactive STAT3 in liver fibrosis. After 2 weeks of administration, we found that Ssb1 and high‐dose S3I‐201 remarkably ameliorated the TAA‐induced morphological changes and liver damage with decreased collagen deposition (Figure S13A). Ssb1 and S3I‐201 decreased the liver/body weight ratio (liver index) and the serum levels of ALT, AST, and TBIL relative to those in the untreated model group (Figure S13B). Biomarkers of liver fibrosis, such as FN, Collagen I, desmin, and α‐SMA, were detected, and 10 mg·kg^−1^ Ssb1 exerted a considerable effect on fibrosis alleviation; which had about the same efficacy as the commercial inhibitor S3I‐201 (Figure 9A; Figure S13C).

Given that the exploration of HSCs has focused on STAT3/Gli1 modulation by Ssb1, we proceeded to examine the regulation in liver tissues. The WB results showed that Ssb1 and S3I‐201 reduced STAT3 phosphorylation and Gli1 expression in mouse liver (Figure 9B; Figure S13D). Co‐staining of p‐STAT3 with α‐SMA demonstrated a decreased accumulation of p‐STAT3 in the aHSCs under Ssb1 or S3I‐201 treatment (Figure S13E). In particular, IF colocalization of Gli1 and p‐STAT3 showed that the interaction and expression of STAT3 and Gli1 were reduced both in the Ssb1 and S3I‐201‐treated groups (Figure 9C). These results collectively demonstrate that Ssb1 attenuated liver fibrosis by targeting STAT3 activation and reducing the interaction of STAT3/Gli1 in the TAA‐induced model.

The antifibrosis efficiency of 10 mg·kg^−1^ Ssb1 was further studied in CCl_4_‐induced liver fibrotic mice. Ssb1 attenuated the fibrosis histopathological characteristics of the liver tissues, similar to its effect on the liver tissues of TAA‐injected mice (Figure S14A). Assessment of the liver function by using the serum levels of ALT, AST, and TBIL revealed that Ssb1 exerted a protection effect like its effect on the TAA model (Figure S14B). Meanwhile, the expression of various proinflammatory cytokines (IL‐6 and IL‐1β) and fibrotic proteins (FN, collagen I, α‐SMA, and vimentin) in the liver tissues was downregulated by Ssb1 treatment (Figure 9D; Figure S14C–E). Ssb1 regulation of p‐STAT3 and Gli1 was also investigated. The results in Figure 9E display an obvious decrease in p‐STAT3 and Gli1 expression levels. We also performed IF colocalization staining of p‐STAT3/α‐SMA and p‐STAT3/Gli1. It was found that Ssb1 decreased STAT3 phosphorylation and Gli1 expression in the aHSCs and blocked the interaction between STAT3 and Gli1 (Figure S14F,G). Overall, the antifibrosis determination in animals showed that Ssb1 could reduce the indices of liver fibrosis in the TAA and CCl_4_‐induced models and regulate STAT3 phosphorylation and p‐STAT3/Gli1 interaction in fibrotic livers.

### 2.11 Knockdown of STAT3 Abolished the Antifibrotic Efficacy of Ssb1

STAT3 was specifically knocked down in mice liver through adeno‐associated virus (AAV) 8‐ApoE‐shSTAT3 to further determine if Ssb1 attenuates liver fibrosis depending on STAT3 (Figure 9F). IF staining of STAT3 showed that the expression of STAT3 was considerably decreased in the liver tissues after AAV injection for 3 weeks (Figure S15A). The co‐staining enhanced green fluorescent protein (EGFP) of AAV8 with the HSC biomarker desmin showed that the EGFP‐positive cells were also positive for desmin, indicating the successful knockdown of STAT3 in the HSCs (Figure S15B).

Liver‐specific STAT3‐KD mice were then used to construct a CCl_4_‐mediated liver fibrosis model for the investigation of Ssb1 antifibrotic efficacy. In mice receiving 3‐weeks CCl_4_ injection, knockdown of STAT3 inhibited the expression of both fibrotic biomarkers and Gli1 compared with the un‐knockdown group, suggesting that STAT3 was important for CCl_4_‐induced liver fibrosis (Figures S15C,D). Ssb1 was then administrated from the 8^th^ week and continued for 4 weeks (Figure 9F). As evidenced by the hematoxylin and eosin, Masson, and Sirius Red staining shown in Figure 9G, knockdown of STAT3 decreased CCl_4_‐induced collagen deposition, and Ssb1 failed to further reduce liver fibrosis in response to CCl_4_ stimulation. The liver index and serum levels of ALT, AST, and TBIL in the Ssb1‐treated shSTAT3 mice were collected for further analysis (Figure 9H,I). Unlike in the un‐knockdown group, Ssb1 treatment no longer decreased the serum levels of ALT, AST, and TBIL in the liver‐specific STAT3‐KD mice. Similarly, in the STAT3‐KD mice, Ssb1 could not further inhibit the CCl_4_‐induced expression of FN, α‐SMA, desmin and collagen production, suggesting that STAT3 knockdown abrogated the antifibrosis effect of Ssb1 (Figure 9J and Figure S15E,F).

Given that STAT3 protein and phosphorylated STAT3 decreased in the shSTAT3 groups, Ssb1 treatment could not further inhibit STAT3 phosphorylation (Figure 9K and Figure S15G,H). The expression of hepatic Gli1 was independent of Ssb1 in the STAT3‐KD mice. Figure 9L indicates that the co‐expression of p‐STAT3 and Gli1 was increased by CCl_4_ induction and decreased by Ssb1 treatment in the normal mice. However, this phenomenon disappeared after the STAT3 knockdown. The mRNA levels of the genes regulated by STAT3, namely, Gli1, displayed a similar tendency as that of the protein expression (Figure S15I). Collectively, these results indicate that STAT3 is required for Ssb1 antifibrosis efficacy and that STAT3 is the target of Ssb1 in the treatment of liver fibrosis.

## 3 Discussion

Stir baking with vinegar as liver medication is a classic theory of Chinese medicine. The contents of Saikosaponin b1 and Saikosaponin b2 were increased, while the Saikosaponin a, Saikosaponin c, and Saikosaponin d were decreased in Radix Bupleuri (Chinese name is Chaihu) after the vinegar‐baking process. Our previous study showed that Ssb1 is a promising antifibrosis compound that is effective in inducing aHSC apoptosis [17]. However, the mechanism through which Ssb1 works remains unclear. In this study, the notion of “chemical target biology in traditional Chinese medicine (TCM) processing” was put forward to pioneer a new scientific direction for TCM integrity and innovation. Based on the above concept, we demonstrated that Ssb1 could directly bind to STAT3 and inhibit its Tyr 705 phosphorylation, dimerization, and nuclear translocation. Transcriptomic analysis showed that Ssb1 decreased the expression of Gli1 by inhibiting STAT3 activation. Meanwhile, Ssb1 competitively prevented STAT3's interaction with Gli1, which inhibited its transcriptional activity and blocked the activation of the Hh signaling pathway. A series of examinations showed that Ssb1 repressed interaction between STAT3 and Gli1, which subsequently promoted Gli1 degradation by the ubiquitin‐proteasome system. As a result, Gli1 degradation regulated the expression of Bcl2 in the Hh signaling pathway and therefore induced aHSCs apoptosis through the mitochondrial pathway (Figure 8E). Ssb1 prevented liver fibrogenesis in the TAA and CCl_4_‐induced animal models by suppressing STAT3 activation and blocking STAT3/Gli1 interaction, and specific knockdown of STAT3 in mice liver further strengthened the critical role of STAT3 in the Ssb1 treatment of liver fibrosis.

STAT3, a member of the STATs family, is a key transcriptional mediator of cellular processes [43, 44]. Activated STAT3 can regulate the transcription of many target genes involved in cell proliferation, migration, differentiation, and apoptosis [28, 45]. Previous studies detected elevated STAT3 phosphorylation in all rodent models with liver injury and fibrosis and in the samples of patients with fibrosis and cirrhosis [46, 47]. Our study revealed that Ssb1 inhibited STAT3 phosphorylation in the activated HSC‐T6 cells without influencing other possible binding proteins, such as PI3K, AKT, and MAPK3 [48, 49]. Biochemical and molecular docking assays demonstrated that Ssb1 bound to STAT3 by directly interacting with S319 residue in CCD. STAT3 has six common structural motifs, namely, NTD, CCD, DBD, the linker domain, the Src homology 2 domain (SH2), and the transactivation domain [50]. CCD has a large hydrophilic surface predominantly involved in the recruitment of STAT3 to its receptor [50], which is essential not only for its activation via SH2 domain‐mediated receptor binding but also for its activation via phosphorylation [51] and nuclear translocation [52]. In our research, Ssb1 reduced STAT3 tyrosine phosphorylation and prevented dimerization and nuclear translocation possibly because Ssb1 directly targeted the S319 residues in CCD. However, the specific modification mechanism needs to be further validated by crystallographic evidence.

Furthermore, we also demonstrated how STAT3 interacted with Gli1 and illustrated the specific function of Ssb1 in inducing aHSCs apoptosis. Our results showed that STAT3 was the upstream of Gli1 and regulated the gene expression of Gli1. Moreover, STAT3 could bind with Gli1 protein on the domain containing SUFU binding sites, which competitively repressed the binding of Gli1 and SUFU, therefore increasing Gli1 stability and nuclear translocation. On the one side, Ssb1 binding with STAT3 inhibited the transcriptional activity of STAT3, which decreased the gene expression of Gli1. On the other hand, Ssb1 binding with STAT3 blocked the interaction between STAT3 and Gli1, which promoted Gli1 binding with SUFU and then impaired Gli1 protein stability by the ubiquitin‐proteasome system. Collectively, Ssb1 abolished Gli1 function by directly binding with STAT3 and decreased Gli1 expression both at the gene level and protein level, resulting in the loss of antiapoptotic mediators and inducing aHSCs apoptosis.

This study has some limitations. This work did not evaluate STAT3 absence in macrophages and hepatocytes in the liver, although STAT3 is a key protein in inflammation. In addition, aside from the regulation of the Hh signaling pathway by inhibiting STAT3/Gli1 interaction, Ssb1 may affect other signaling pathways by directly binding with STAT3. This topic was not investigated in this study, and it needs to be explored in the future.

In summary, this study identified Ssb1 as a novel STAT3 inhibitor in liver fibrosis treatment. Ssb1 directly binds to STAT3, suppresses the activation of STAT3, and induces aHSCs apoptosis by regulating the STAT3/Gli1/Bcl2 axis in the Hh signaling pathway. This study also revealed the activities of TAA‐ or CCl_4_‐induced liver fibrosis in mice. Overall, Ssb1 as a promising compound needs to be developed further for the clinical therapy of hepatic fibrosis.

## Author Contributions

Conceptualization: Meiyu Shao, Mengyun Peng, and Gang Cao. Investigation: Meiyu Shao, Xiaoqing Zhang, Hongyan Dong, and Jiamei Sun. Methodology: Xin Han, Qiao Yang, and Lu Wang. Visualization: Meiyu Shao and Xiaoqing Zhang. Software: Roufen Chen, Liteng Shen, and Lei Xu. Resources: Dongxin Tang, Shuosheng Zhang, and Keda Lu. Revised experiments: Meiyu Shao and Jiamei Sun. Writing—original draft: Meiyu Shao and Mengyun Peng. Writing—revise and editing: Meiyu Shao, Mengyun Peng, Bo Zhu, and Gang Cao. Supervision: Mengyun Peng and Gang Cao.

## Conflicts of Interest

The authors declare no conflicts of interest.

## Supporting information



Supporting Information

## Data Availability

Data supporting the results in this work are available from the corresponding authors with reasonable request. The transcriptome sequence data have been submitted to the Sequence Read Archive (SRA) databases under BioProject number PRJNA1119293.
