# Supplementary material for: Saikosaponin b1 Attenuates Liver Fibrosis by Blocking STAT3/Gli1 Interaction and Inducing Gli1 Degradation
Source: Exploration (Beijing). 2025 Feb 3;5(5):70000. doi: 10.1002/EXP.70000 (PMC12561301; doi:10.1002/EXP.70000)
Supplement: Supplementary file 1 — Supporting Information [file EXP2-5-70000-s001.docx]

**Supplementary Information**

**for**

**Saikosaponin b1 attenuates liver fibrosis by blocking STAT3/Gli1 interaction and inducing Gli1 degradation**

Meiyu Shao^#,1^, Xiaoqing Zhang^#,1^, Jiamei Sun^#,1^, Hongyan Dong^1^, Xin Han^1^, Qiao Yang^1^, Roufen Chen^2^, Liteng Shen^2^, Lei Xu^3^, Lu Wang^1^, Bo Zhu^1,4^, Dongxin Tang^5^, Shuosheng Zhang^6^, Keda Lu^7^, Mengyun Peng*^,1^, Gang Cao*^,1,7^

^1^School of Pharmacy, Zhejiang Chinese Medical University, 310053 Hangzhou, P. R. China.

^2^Innovation Institute for Artificial Intelligence in Medicine, Zhejiang University, Hangzhou 310058, China.

^3^Institute of Bioinformatics and Medical Engineering, School of Electrical and Information Engineering, Jiangsu University of Technology, Changzhou, 213001, P.R. China.

^4^Microbiology and Genetics Department, University of Salamanca, Salamanca 37007, Spain.

^5^Department of Science and Education, The First Affiliated Hospital of Guiyang University of Chinese Medicine, 550001 Guiyang, China

^6^College of Chinese Materia Medica and Food Engineering, Shanxi University of Chinese Medicine, Jinzhong, 030600, China

^7^The Third Affiliated Hospital of Zhejiang Chinese Medical University, Hangzhou, China

^#^These authors contributed equally to this work.

*Corresponding author. E-mail address: pmy@zcmu.edu.cn; caogang33@163.com.

**1. Experimental Section**

*Ethics:* All experiments of animals have received the approval of Animal Ethical and Welfare Committee of Zhejiang Chinese Medical University (Hangzhou, China, Project no. IACUC-20231113-02 and IACUC-20231113-03). Human liver samples were obtained from The First Affiliated Hospital, Guizhou University of Traditional Chinese Medicine, and were approved by the Ethics Committee of the First Affiliated Hospital of Guizhou University of Traditional Chinese Medicine (Project no. K2021-066).

*Western blot (WB) analysis:* Total proteins were separated by electrophoresis with 8%–10% sodium dodecyl sulfate-polyacrylamide gel electrophoresis (SDS-PAGE) and electro transferred to polyvinylidene fluoride membranes. Then, the membranes were blocked with 5% non-fat milk in PBST for 1 h and incubated with primary antibodies overnight at 4 ℃. The membranes were washed with PBST and treated with secondary antibodies for 1 h at room temperature. Immunoreactive bands were visualized using a protein image system (Protein sample, FE1216), and signals were analyzed using Image J.

*Reverse transcription-quantitative PCR (RT-qPCR):* Total RNAs were isolated using the Eastep Super Total RNA Extraction Kit (Promega, China) and reversely transcribed into cDNA using the Yfx Script First Strand cDNA Synthesis Kit (YIFEIXUE BIO TECH, China). Realtime quantitative PCR using SYBR Green Master Mix was performed with Light-Cycler 96 System (Roche Diagnostics GmbH). The quantification and concentration of RNA were measured using the NanoDrop 2000 Spectrophotometer (Thermo Fisher Scientific, USA). The following specific primer sequences were used: Relative expression was calculated using the comparative threshold cycle method and determined by using the value of 2^−ΔΔCt^. The primers used are listed in Supplementary Table.

*Immunofluorescence (IF) experiments:* The cells were washed three times with precooled PBS, fixed in 4% paraformaldehyde for 40 min, and then permeabilized with 0.1% Triton X-100 for 10 min. After washing with PBS, the cells were blocked in 5% goat serum for 30 min at room temperature. Following the removal of the blocking buffer, cells were incubated with primary antibodies overnight at 4 ℃. On the next day, the cells were washed with PBS prior to incubation with secondary antibodies for 1 h at room temperature. After staining the cells with DAPI, fluorescent images were taken using a fluorescence microscope (ZEISS, AXIO SCOPE.A1).

*Proteome microarray assays:* Protein binding microarray chips were obtained from the Johns Hopkins Medical Institutions Protein Microarray Core (CDI Laboratories, Inc.). The experiments and data processing were performed by Wayne Biotechnology Company (Shanghai, China). In brief, HuProt proteome microarrays were blocked with a blocking buffer. Then, biotin-Ssb1 in a blocking buffer (10 µmol·L^-1^) was incubated with the blocked proteome microarrays at room temperature for 1 h. Thereafter, the microarrays were washed, and Cy5-streptavidin was added. Finally, the microarrays were scanned using the Axon GenePix 4000B. GenePix Pro 6.0 (Axon Instruments) was used to extract data from the recorded microarray images. Protein spots with a signal intensity indicator Z-score of >2.8 in Ssb1-bio-treated microarrays and <2.8 in biotin-treated microarrays were identified as candidate positive proteins. Enrichment analysis, including the Kyoto Encyclopedia of Genes and Genomes (KEGG) pathway, was performed using the clusterProfiler package in RStudio.

*Surface plasmon resonance (SPR) assay:* The binding affinity of Ssb1 to recombinant human STAT3 (rhSTAT3) was determined using OpenSPR TM (Nicoya) equipped with a NTA sensor chip (SNE011). The rhSTAT3 protein was dissolved in 40 μg·mL^-1^ of ligand buffer, and then the buffer was injected to the sample channel for 240 s at a flow rate of 20 μL·min^-1^. Different concentrations of Ssb1, including 80, 40, 20, 10, 5, and 0 μM, were prepared using a running buffer (PBS containing 1% DMSO). The sensor chip surface should be regenerated completely with 10 mM glycine-HCl as injection buffer to remove the analyte, and then the next concentration cycle of the analyte Ssb1 must repeat the injection and regeneration steps. The dissociation constant (KD values) of the GAamide–protein complex was calculated using TraceDrawer (Ridgeview Instruments ab, Sweden).

*Cellular thermal shift assay (CETSA):* For dose–response CETSA experiment, the cell lysates were obtained by freeze thawing five times in liquid nitrogen. Cell lysates were divided into seven aliquots, with one aliquot used as control and the others being incubated with different concentrations of Ssb1 for 3 min at room temperature. The heating time and temperature were kept constant (37 ℃, 52 ℃, 57 ℃). For the CETSA melt curve, cell lysates were divided into two aliquots, with one aliquot used as control and the other being incubated with Ssb1 (5 μmol·L^-1^) for 5 min at room temperature. Then, the lysates were heated individually at indicated temperatures (37 °C–67 ℃) for 3 min, followed by cooling with ice. The soluble lysates were centrifuged at 12,000 g for 20 min at 4 ℃, and then supernatants were analyzed by SDS-PAGE and immunoblotting.

*Pull-down assay:* A biotinylated protein interaction pull-down kit was utilized to examine the Ssb1–STAT3 interaction. 50 mM biotinylated-Ssb1 and 40 μL of streptavidin-agarose beads were added to 500 μL of cell lysates. Biotin or Ssb1 alone was used as a control. Lysates were prepared from HSCs and liver tissues. The mixture was incubated at 4 °C for 24 h with gentle rocking. Then, the samples were spun and washed three times. Eluent buffer was boiled with 1× loading buffer. The final samples eluted were loaded onto a 10% polyacrylamide gel for WB.

*Identification of the Ssb1-binding site:* The online deep-learning-based prediction tool, DeepSite (https://open.playmolecule.org/tools) to identify potential small-molecule binding pockets of the STAT3 protein. DeepSite uses a three-dimensional convolutional neural network (3D-CNN) to voxelize the protein’s spatial structure trained on a large set of known protein–ligand complexes, allowing it to predict latent ligand-binding sites of protein. For STAT3, DeepSite outputs the Cartesian coordinates of the center of each candidate pocket together with a confidence score (Score); higher scores indicate greater reliability. We select four candidate centers with scores ≥ 0.89 for further evaluation, and validated their feasibility by structural alignment and molecular docking: Site 1: Score = 0.9981, Center = (21.87, 11.39, –39.03); Site 2: Score = 0.9882, Center = (–0.13, –6.61, 4.97); Site 3: Score = 0.9764, Center = (–10.13, 31.39, –1.03); Site 4: Score = 0.8910, Center = (–40.13, –8.61, 46.97).

To assess the functional relevance of the predicted pockets, we performed spatial-proximity analyses. For each DeepSite center, a 20 Å radius sphere was constructed, and a Biopython script was used to filter the AlphaFold2 predicted STAT3 crystal structure (AF-P40763-F1) at the atomic level. Euclidean distance from every atom of the residue—both backbone and side-chain atoms—to the pocket center was calculated. If any atoms lay within 20 Å or less, the residue was deemed to be in the vicinity of the site. And there are key findings of the predicted residues of STAT3 protein: two important residues were identified around Site 2 (–0.13, –6.61, 4.97), Ser319 (chain A, 18.82 Å) as a peripheral modulatory residue and Arg325 (chain A, 16.97 Å) as a direct binding residue of ligand were predicted; near Site 3 (–10.13, 31.39, –1.03), Asp371 (chain A, 11.67 Å) was identified, which located close to the protein surface and possessed the structural features for charge-based ligand recognition. For Site 1 and Site 4, no functionally relevant residues reported in the literature were found within the 20 Å neighborhood.

*Molecular docking:* The structure of the STAT3 protein (AF-P40763-F1) was derived from the AlphaFold Protein Structure Database1.2. The 3D structure of saikosaponin B1 was sketched using the builder panel in Maestro. Then, the LigPrep module of the Schrödinger 2021-2 suite, with default settings, was used to process all ligands. The 3D grid boxes of 10 Å were created and centered on the residues Y94, S319, R325, and D371. Subsequently, the pre-processed ligands were docked into the study model using the Schrödinger induced fit docking (IFD) protocol with extended sampling. In adjusting the receptor and ligand action potentials, we applied a scaling factor of 0.5 and selected the docking precision (XP), retaining a maximum of 20 poses. The IFD scores were calculated by amalgamating the protein–ligand interaction energy and the system-wide energy. Leveraging these XP docking and IFD scores, we ranked the results and identified the most optimal complex conformation for subsequent molecular dynamics (MD) simulations. Then, MMGBSA was used to calculate the binding free energy of small molecules to proteins or macromolecules. It relies on MD simulations of the receptor–ligand complexes. The binding free energy of the complex was estimated by considering the free energies of the ligand and protein separately:

ΔG_Binding_ = ΔG_Complex_−ΔG_Protein_−ΔG_Ligand_

*Molecular dynamics:* Molecular dynamics simulations were utilized to examine the complex conformation of the STAT3/S319-Ssb1 complex in detail. The initial conFigureuration for MD simulations was derived from IFD. The simulations were set up using Amber software, utilizing the ff19sb protein force field and the GAFF small molecule force field as fundamental parameters. The complex molecules were automatically placed at the center of a cubic box surrounded by TIP3P water molecules. Simulations were conducted under isothermal–isobaric (NPT) conditions at a constant temperature of 300 K, with the box boundaries maintained at a minimum distance of 10 Å from the farthest point of the protein to ensure a sufficient water buffer. In neutralizing the overall charge of the system, 0.15 M sodium chloride was added, along with minor amounts of Na^+^ and Cl^−^ ions. Prior to the simulation, energy minimization was performed using steepest descent and conjugate gradient methods to alleviate any steric clashes. After preparing and energy minimizing the model, it was input into the MD module for 50 ns simulation, with trajectory data recorded at 1 ns intervals. The simulations were executed at a constant temperature of 300 K and a pressure of 1 atm (301.01325 bars) using a Berendsen thermostat and barostat. A uniform force field was applied throughout the simulations.

*Histological analysis:* Paraffin-embedded human liver tissues were rehydrated and then stained with hematoxylin and eosin (H&E). Sections were stained with Masson’s Trichrome and Sirius Red to monitor collagen distribution. The fibrotic areas were calculated by Image J as the summation of blue-stained areas in Masson staining divided by the total liver area. The liver was evaluated in accordance with the following scoring system: F0: no liver fibrosis; F1: 10% or fewer fibrotic areas; F2: 11%–20% fibrotic areas; F3: 21%–30% fibrotic areas; F4: 31% or more fibrotic areas.

*STAT3 knockdown and expression:* Gene silencing in cells was achieved by transfecting cell shRNA. pLKO.1-shSTAT3 was provided by Tsingke Biotech. Control cells were transfected with negative control shRNA. HSC-T6 were transfected using Lipofectamine 3000 (Thermo Fisher, Carlsbad, CA). STAT3 overexpression in cells was achieved by transfecting 293T cells with STAT3 plasmids (pCDNA3.1-ratSTAT3-flag, Tsingke Biotech Inc., Beijing, China). In addition, mutant STAT3 plasmids (pCDNA3.1-ratSTAT3-flag-S319A, and pCDNA3.1-ratSTAT3-flag R325A, D371A) were designed and obtained from Tsingke Biotechnology Co., Ltd.

*Knockout of STAT3 by CRISPR-Cas9:* Targeting sequences were designed at CRISPR direct (http://chopchop.cbu.uib.no/). The knockout STAT3 HSC-T6 was generated by CRISPR-Cas9 targeting the following site: 5′-aggtctcaagatcgacctagagg-3′. HSC-T6 were transfected with plasmids using Lipofectamine 3000 in accordance with the manufacturer’s instruction. After transfection for 48 h, cells were selected for 2 days in the presence of 5 mg·mL^-1^ of puromycin. The gene knockout clones were confirmed by immunoblotting.

*Transient transfection:* HEK-293T cells were seeded in a dish (6 cm i.d.) overnight. STAT3-HA and STAT3-Flag plasmids obtained from Tsingke were co-transfected with Lipofectamine 3000 for 48 h. Recombinant plasmids encoding human full-length STAT3, Myc tagged truncated Gli1 were obtained from Genechem (Shanghai, China).

Cells were treated with Ssb1 (10 μM) or S3I-201 (50 μM) and then stimulated with IL-6 (20 ng·mL^-1^) for 24 h before harvest. Then cells were collected and lysed in NP40 lysis buffer and then incubated with an antibody or IgG as a negative control with rotation overnight at 4 °C. Afterward, the cells were added to 10 μL of protein A/G magnetic beads, shaken for 6 h at 4 °C, and washed five times following with lysis buffer. Immunoprecipitation complexes were eluted with a loading buffer and detected by WB.

*Dual-luciferase screening assay:* HEK-293T cells were plated in 24-well culture plates and co-transfected transiently with the pSTAT3-TA-luc (Beyotime) and Renilla plasmids using Lipofectamine 3000 (Invitrogen, Carlsbad, CA, USA). After transfection for 24 h, the cells were stimulated with recombinant human IL-6 (20 ng·mL^-1^) and treated with Ssb1 (1, 5, and 10 μM) for 24 h. Afterward, the cell lysates were prepared, and the luciferase activity was detected using the Dual-Luciferase Reporter kit (Beyotime, RG088S). The data were expressed as the ratio of Firefly to Renilla luciferase activity.

*JC-1 staining:* Mitochondrial membrane depolarization was monitored on the basis of the changes in the tetraethyl-benzimidazolyl carbocyanine iodide (JC-1, Beyotime, green: red fluorescence ratio), where an increased ratio indicates an increase in mitochondrial membrane potential (MMP). Moreover, the increased ratio may imply the early stage of apoptosis. Cells were incubated with JC-1 (1:5,000 dilution) for 30 min at 37 °C. Then, the cells were washed two times with 1× PBS. The fluorescence intensity was measured by using a fluorescence microscope.

*Terminal deoxynucleotidyl transferase dUTP nick-end labeling (TUNEL) assay and Annexin V-FITC apoptosis detection:* TUNEL staining was performed in accordance with the TUNEL assay kit (Beyotime) protocol. The cells were stained with DAPI for nuclear, then observed under a fluorescence microscope.

HSC-T6 cells were treated with or without Ssb1 for 24 h, then cells were digested with EDTA-free trypsin. After collecting and washing trice with PBS, cells were incubated with Annexin V/PI kit (BD Biosciences) according to the manufacture’s protocol in the dark and analyzed by flow cytometer in darkness. Cells which stained positive for Annexin V and PI were recognized as late apoptosis.

*Animal experiments:* Balb/c mice were maintained in the center for Experimental Animals at Zhejiang Chinese Medical University, Zhejiang, China. All experiments were performed in accordance with the protocols approved by the Committee for Animal Research of Zhejiang Chinese Medical University and in accordance with the University of Health Guide for the Care and Use of Laboratory Animals. Male Balb/c mice aged 6–8 weeks (body weights of 18–20 g) were purchased from Shanghai SLAC Laboratory Animal Co., Ltd. (Shanghai, China). The ethic number is IACUC-20231113-02 and IACUC-20231113-03. Mice were randomly assigned to different treatment groups. All animals were maintained under constant humidity and temperature and kept on a 12 h light, 12 h dark cycle with free access to food and water.

*Thioacetamide (TAA)-**induced fibrosis model:* After 1-week acclimatization, the male Balb/c mice (body weight of 18–20 g) were randomly divided into seven groups and intraperitoneally (i.p.) injected with 100 mg·kg^-1^ of TAA three times a week in the first week, then intraperitoneal injected with 200 mg·kg^-1^ of TAA twice a week for another 5 weeks ^[2, 3]^. Mice were also treated with PBS, Ssb1 (5, 10, 20 mg·kg^-1^), or S3I-201 (5, 10 mg·kg^-1^) three times a week from week 4 to week 7 (i.p., n=6 per experimental group). Animals were sacrificed 24 h after the final treatment. All animal procedures were approved by the Animal Care and Use Committee of Zhejiang Chinese Medical University.

*Chronic carbon tetrachloride (CCl_4_)-induced the fibrosis model:* After 1-week acclimatization, Balb/c mice were treated once with subcutaneous injection of 5 mL·kg^-1^ of 40% CCl_4_ in the first week, then subcutaneously injected with 3 mL·kg^-1^ of 30% CCl_4_ twice a week for another 6 weeks to induce liver fibrosis. After two weeks injection of 30% CCl_4_, 10 mg·kg^-1^ of Ssb1 was administered twice a week for 4 weeks ^[4, 5]^. Animals were sacrificed 24 h after the final treatment (n=4 per experimental group).

*Mice infected with adeno-associated virus (AAV):* For STAT3 knockdown experiments in vivo, AAV8 encoding a shRNA under the control of the ApoE/hAATp-promoter, targeting murine STAT3, was provided by GeneChem (Shanghai, China). pAAV8-ApoE/hAATp-EGFP-STAT3-shRNA or a respective control virus (pAAV8-ApoE/hAATp-EGFP-control-shRNA), containing 1 × 10^11^ v.g. in 0.15 mL of saline solution, was intravenously injected into the tail vein of each mouse for liver-specific STAT3 knockdown. After injection AAV8 for 3 weeks, 2 mice were picked randomly to investigate the STAT3 knockdown in liver.

Mice were then treated with CCl_4_ to from the 5th week induce liver fibrosis with the same scheme as before. After two weeks injection of 30% CCl_4_, 20 mg·kg^-1^ of Ssb1 was administered twice a week for 4 weeks. Animals were sacrificed after the final treatment (n = 6 per experimental group).

*Biochemical and histological assays:* The whole blood was left for 30 min at room temperature for complete clot formation. All were then centrifuged at 3000 rpm for 15 minutes to get the serum. Alanine aminotransferase (ALT), aspartate transaminase (AST), and total bilirubin (TBIL) levels were detected in the serum by using an automatic biochemical analyzer (Beckman Coulter, Miami, FL.USA). Liver tissues were fixed, embedded, sectioned, stained with H&E for histological examination, and stained with Masson and Sirius Red. Liver tissue samples were examined under a light microscope (Nikon, E200) for each microscopic field.

*Antibodies:* The primary antibodies used in this study were as follows: anti-STAT3 (Proteintech, 10253-2-AP, 1:3000 for IB, 1:200 for IP), anti-STAT3 (Abcom, ab68153, 1:200 for IF), anti-pSTAT3 Y705 (CST, 9145T, 1:2000 for IB, 1:200 for IF), anti-pSTAT3 S727 (Abcom, ab32143, 1:2000 for IB), anti-α-smooth muscle actin (Proteintech, 14395-1-AP, 1:1000 for IB), anti-α-SMA (ab7817, 1:500 for IF), anti-Collagen I (Proteintech, 14695-1-AP, 1:1000 for IB), anti-Fibronectin (FN; Abcom, ab2413, 1:1000 for IB), anti-Desmin (Abcom, ab8592, 1:1000 for IB and 1:200 for IF), anti-Vimentin (Abcom, ab92547, 1:1000 for IB), anti-GAPDH (Proteintech, 60004-1-Ig, 1:60000 for IB), anti-HIF-1α (Affinity, AF1009, 1:1000 for IB), anti-MAPK3 (CST, cs4695, 1:1000 for IB), anti-p-MAPK3 (CST, cs4370, 1:1000 for IB), anti-PI3K (Proteintech, 67071-1-Ig, 1:1000 for IB), anti-p-PI3K (CST, 17366s, 1:1000 for IB), anti-AKT1 (bs,0115R, 1:1000 for IB), anti-p-AKT1 (Thr34) (bs,5194R, 1:1000 for IB), anti-α-Tubulin (Proteintech, 11224-1-AP, 1:5000 for IB), anti-PFKFB3 (Huabio, ET1705-66, 1:500 for IB), anti-Flag-tag (Proteintech, 20543-1-AP, 1:3000 for IB and 1:200 for IP), anti-HA-tag (Beyotime, AH-158, 1:1000 for IB and 1:100 for IP), anti-Myc (Proteintech, 60003-2-Ig, 1:2000 for IB), anti-Gli1 (Proteintech, 66905-1-Ig, 1:2000 for IB and 1:200 for IP), anti-bcl2 (Abcom, ab194583, 1:1000 for IB), anti-rabbit IgG (CST, 2729s, 1:200 for IP), anti-mouse IgG (Proteintech, B900620, 1:200 for IB), Goat Anti-Rabbit IgG H&L (HRP; Abcom, ab97051, 1:5000 for IB), Goat Anti-Mouse IgG H&L (HRP; Boster, BA1050, 1:5000 for IB), Alexa Fluor 488 goat anti-rabbit IgG (Beyotime, A0423, 1:200 for IF), CY3-conjugated goat anti-Rabbit IgG (Affinity, S0011, 1:100 for IF), and Alexa Fluor 647 goat anti-mouse IgG (Abcom, ab150115, 1:100 for IF).

*Statistics:* Statistical analysis was performed using GraphPad prism version 8. Statistical significance was tested by using the Student’s t-test or one-way ANOVA. The quantitative data were presented as mean ± standard deviation (SD) and as the number (percent) for categorical variables. All tests were two sided, and *P* < 0.05 was considered statistically significant.

**References**

[1] A. T. Kong, F. V. Leprevost, D. M. Avtonomov, D. Mellacheruvu, A. I. Nesvizhskii, *Nat. Methods* **2017**, *14*, 513-520.

[2] X. W. Zhang, J. C. Zhou, D. Peng, F. Hua, K. Li, J. J. Yu, X. X. Lv, B. Cui, S. S. Liu, J. M. Yu, F. Wang, C. C. Jin, Z. N. Yang, C. X. Zhao, X. Y. Hou, B. Huang, Z. W. Hu, *Autophagy* **2020**, *16*, 782-796.

[3] H. H. Wang, J. H. Huang, M. H. Sue, W. C. Ho, Y. H. Hsu, K. C. Chang, M. S. Chang, *EBioMedicine* **2021**, *64*, 103213.

[4] A. M. Chow, M. Tan, D. S. Gao, S. J. Fan, J. S. Cheung, Z. Qiao, K. Man, Z.-R. Lu, E. X. Wu, *Invest. Radiol.* **2013**, *48*, 46-54.

[5] B. Shen, L. Deng, Y. Liu, R. Li, C. Shen, X. Liu, Y. Li, H. Yuan, *Chin. Herb. Med.* **2022**, *14*, 104-110.

**2. Supporting Figures**


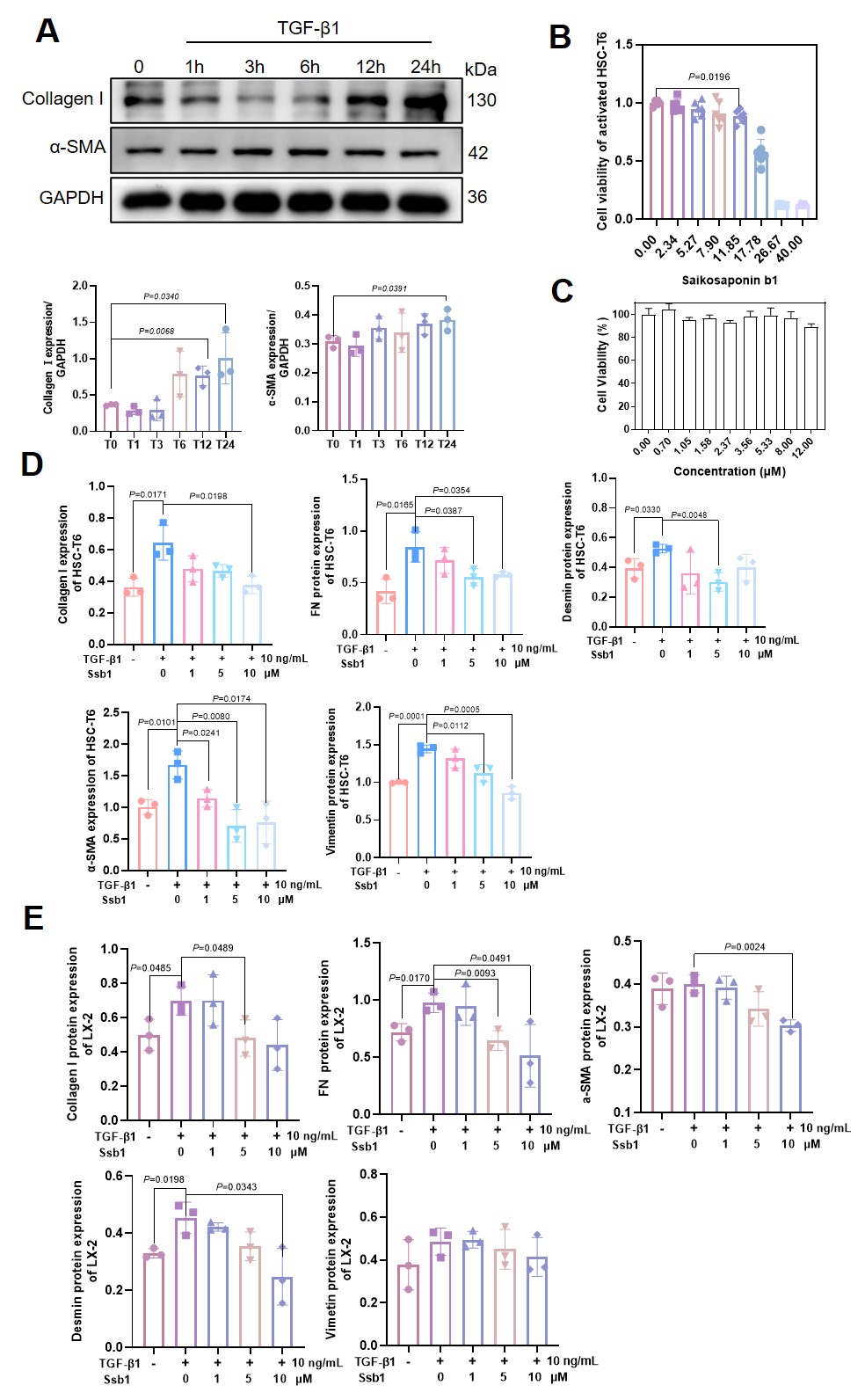


**Figure S1.** (A) Time course of Collagen I and α-SMA induction in response to TGF-β1 in HSC-T6. Cells were exposed to 10 ng·mL^-1^ of TGF-β1 for 24 h. (B, C) Cytotoxicity of Ssb1 in activated HSC-T6 and normal BRL-3A cell lines using MTT assay. (D, E) Densitometric quantification of protein levels in immunoblots presented in Figure 1D and 1E. All statistical data were presented as mean ± SD. Unpaired two-tailed t-test.


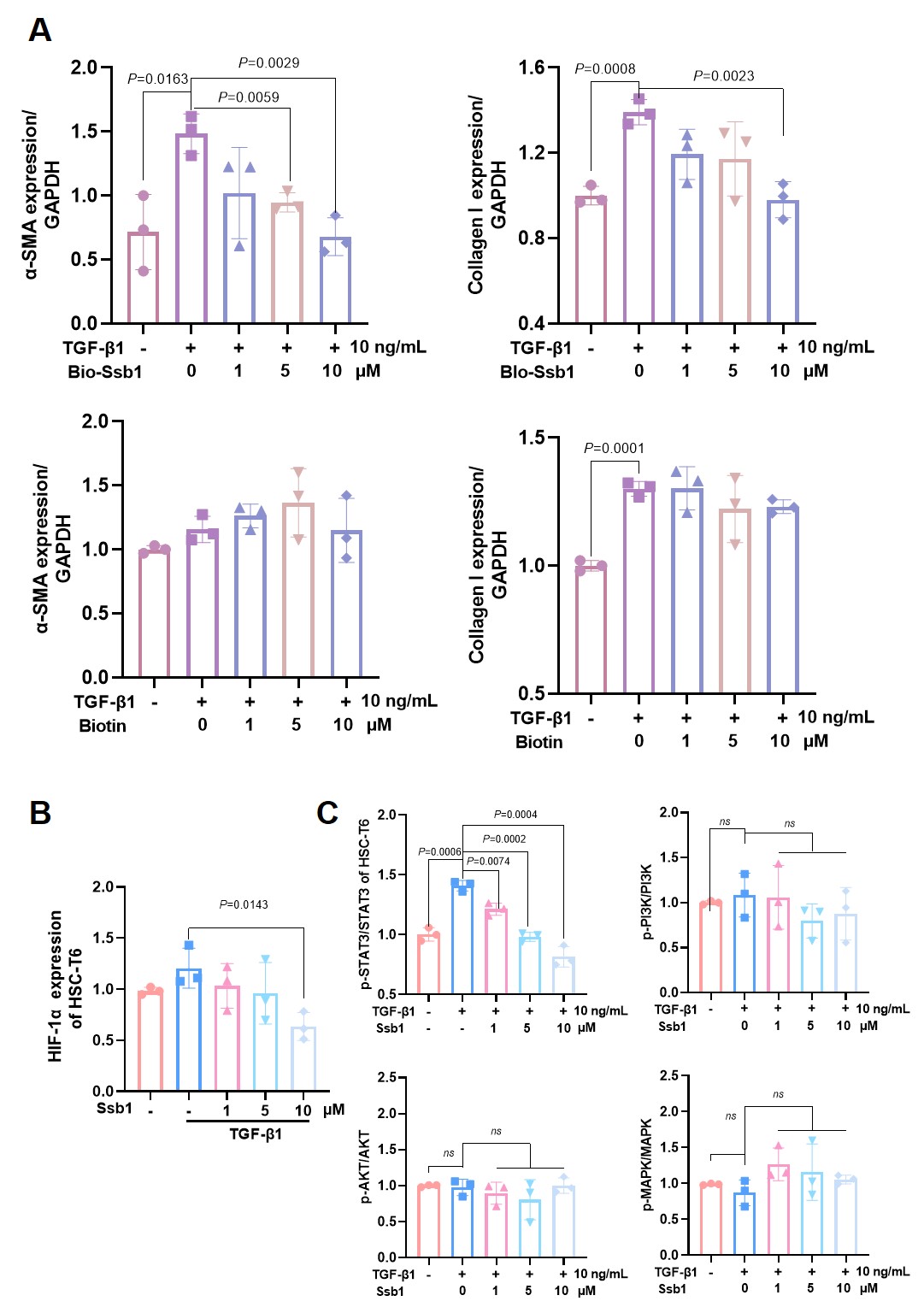


**Figure S2.** (A) Densitometric quantification of protein levels in immunoblots presented in Figure 2C. (B) Densitometric quantification of protein levels in immunoblots presented in Figure 2H. (C) Densitometric quantification of protein levels in immunoblots presented in Figure 2I.


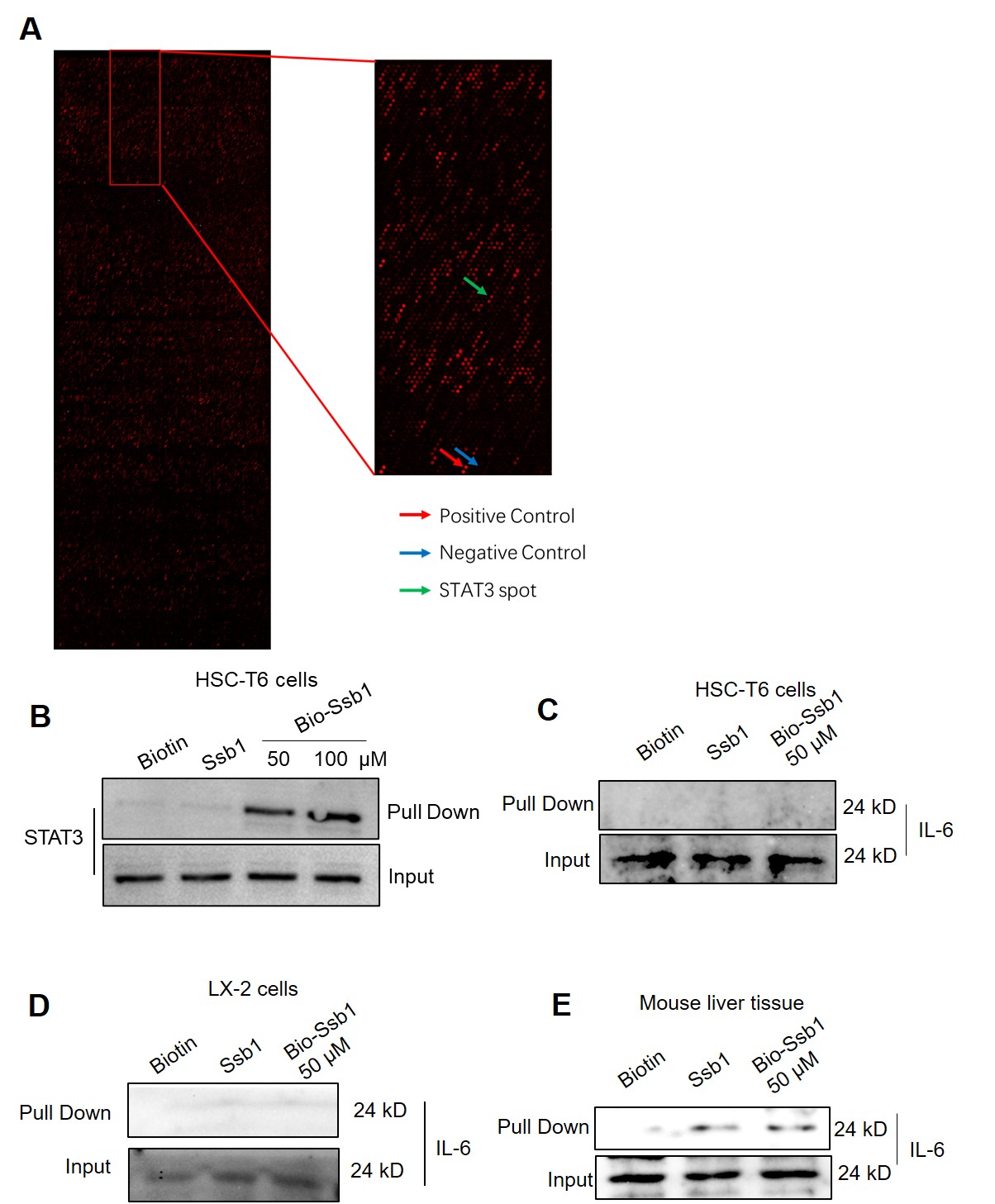


**Figure S3.** (A) Representative image of protein array showing positive red arrow and negative control blue arrow spots, as well as spots for STAT3 green arrow. (B) Biotinylated Ssb1 (Bio-Ssb1) was added to streptavidin–agarose beads and incubated. Biotin or Ssb1 alone was used as a control. Lysates prepared from HSC-T6 were added to the streptavidin–agarose beads with Bio-Ssb1. Then, an eluent was loaded onto a polyacrylamide gel for WB analysis. (C–E) Lysates prepared from HSC-T6 (C), LX-2 (D), and mouse liver tissues (E) were examined for Ssb1 binding to IL-6 using pull-down assay.


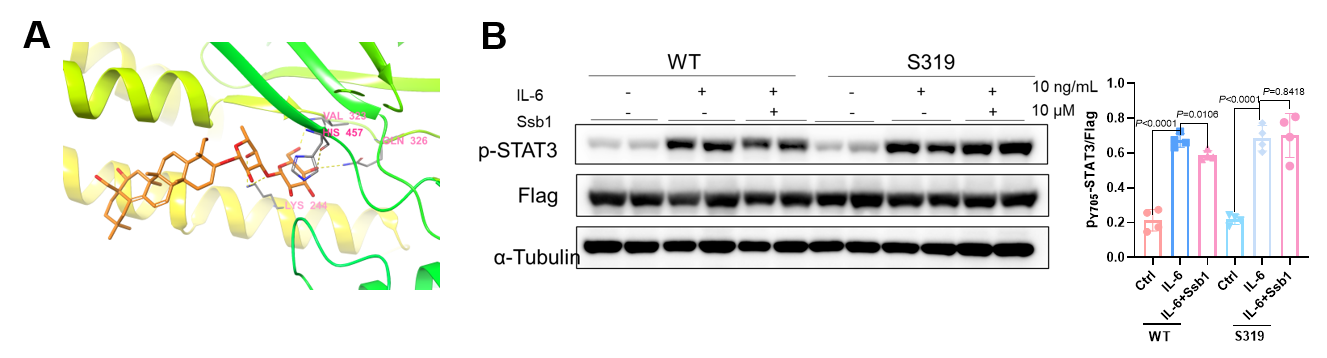


**Figure S4.** (A) 3D diagram representing the protein–ligand interaction in the Ssb1 complex with STAT3-S319. (B) WB repeat of S319A mutation blocked the inhibitory effect of Ssb1 on STAT3 phosphorylation.


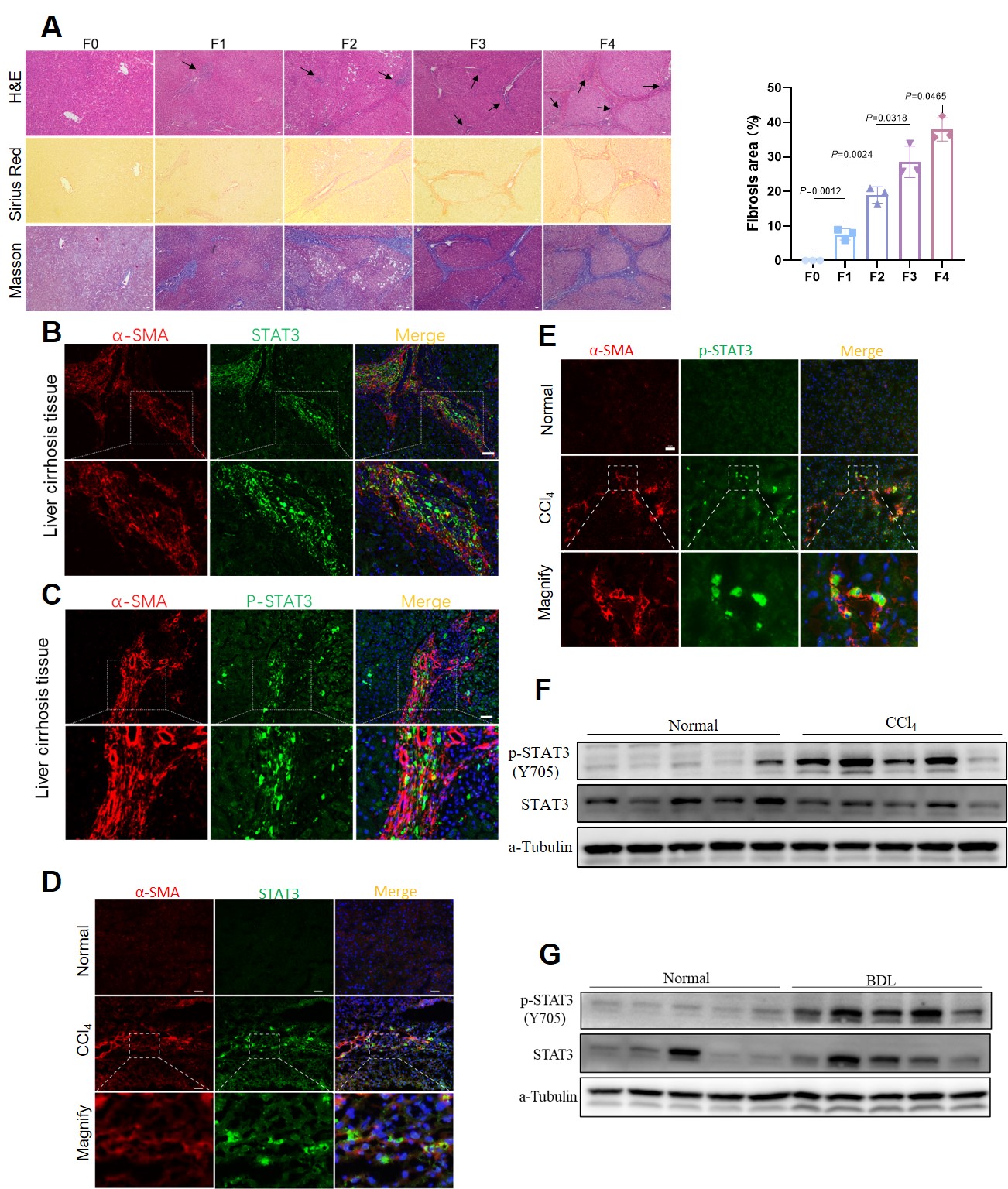


**Figure S5.** (A) Representative images of clinical human liver tissues stained with H&E, Sirius Red, and Masson with different stages of liver fibrosis. Scale bars, 50 μm. (B–C) Dual immunofluorescence staining analysis of STAT3 (B) or p-STAT3 (C) with α-SMA in liver cirrhosis tissue. Scale bars, 50 μm. (D–E) Dual immunofluorescence staining analysis of STAT3 (D) or p-STAT3 (E) with α-SMA in CCl_4_-induced fibrotic liver. Scale bars, 50 μm. (F–G) Protein levels of p-STAT3 were detected in CCl_4_ (F) or bile-duct-ligation (BDL) (G) induced fibrotic liver.


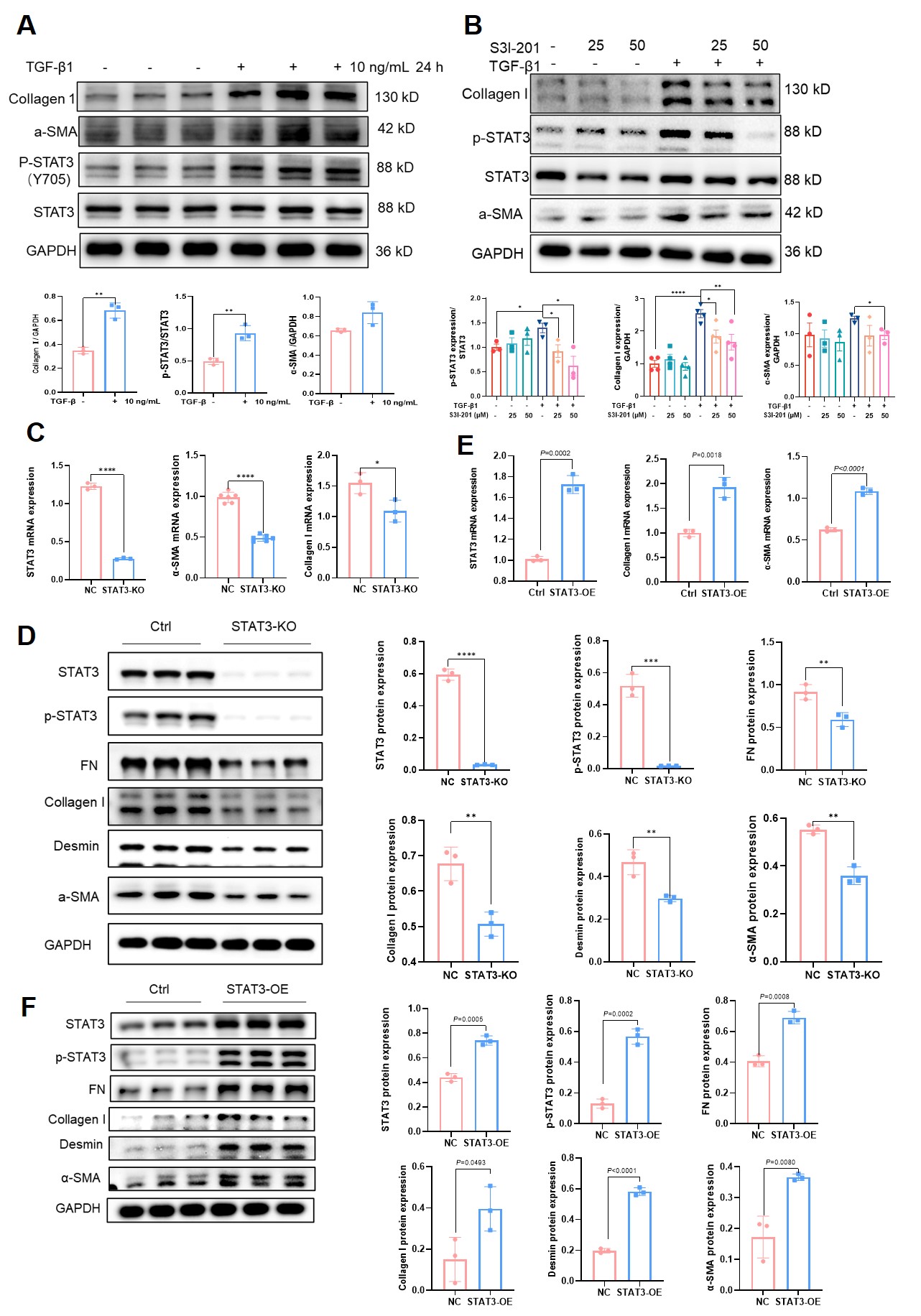


**Figure S6.** STAT3 Activation in HSCs. WB analysis of the expression of α-SMA, collagen I, p-STAT3, and STAT3 in HSC-T6 (A) treated with TGF-β 10 ng·mL^-1^ for 24 h or (B) treated with different concentrations of S3I-201 for 24 h. (C) mRNA levels of Collagen I and α-SMA in STAT3-knockout HSC-T6. (D) Representative immunoblotting images of FN, Collagen I, α-SMA, and Desmin in STAT3-knockout HSC-T6. (E) mRNA levels of Collagen I and α-SMA in STAT3-overexpressed HSC-T6. (F) Protein levels of FN, Collagen I, α-SMA, and Desmin in STAT3-overexpressed HSC-T6.


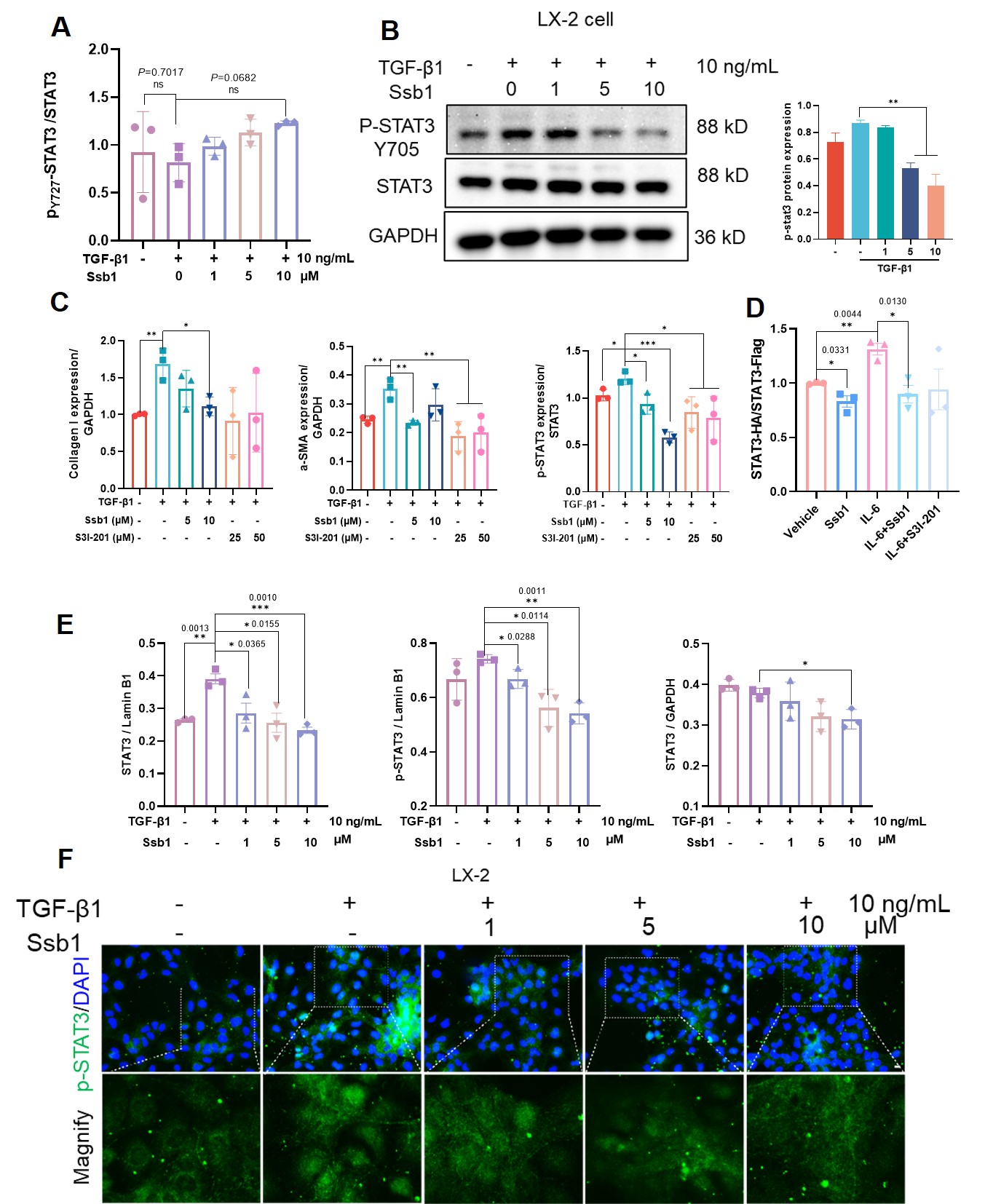


**Figure S7.** (A) Densitometric quantification of protein levels in immunoblots presented in Figure 5C. (B) Ssb1 inhibits TGF-β1-induced STAT3 phosphorylation Tyr-705 in LX-2 cells. (C) Densitometric quantification of protein levels in immunoblots presented in Figure 5D. (D) Densitometric quantification of protein levels in immunoblots presented in Figure 5E. (E) Densitometric quantification of protein levels in immunoblots presented in Figure 5F. (F) IF staining analysis of p-STAT3 in LX-2 cells. Scale bars, 10 μm.


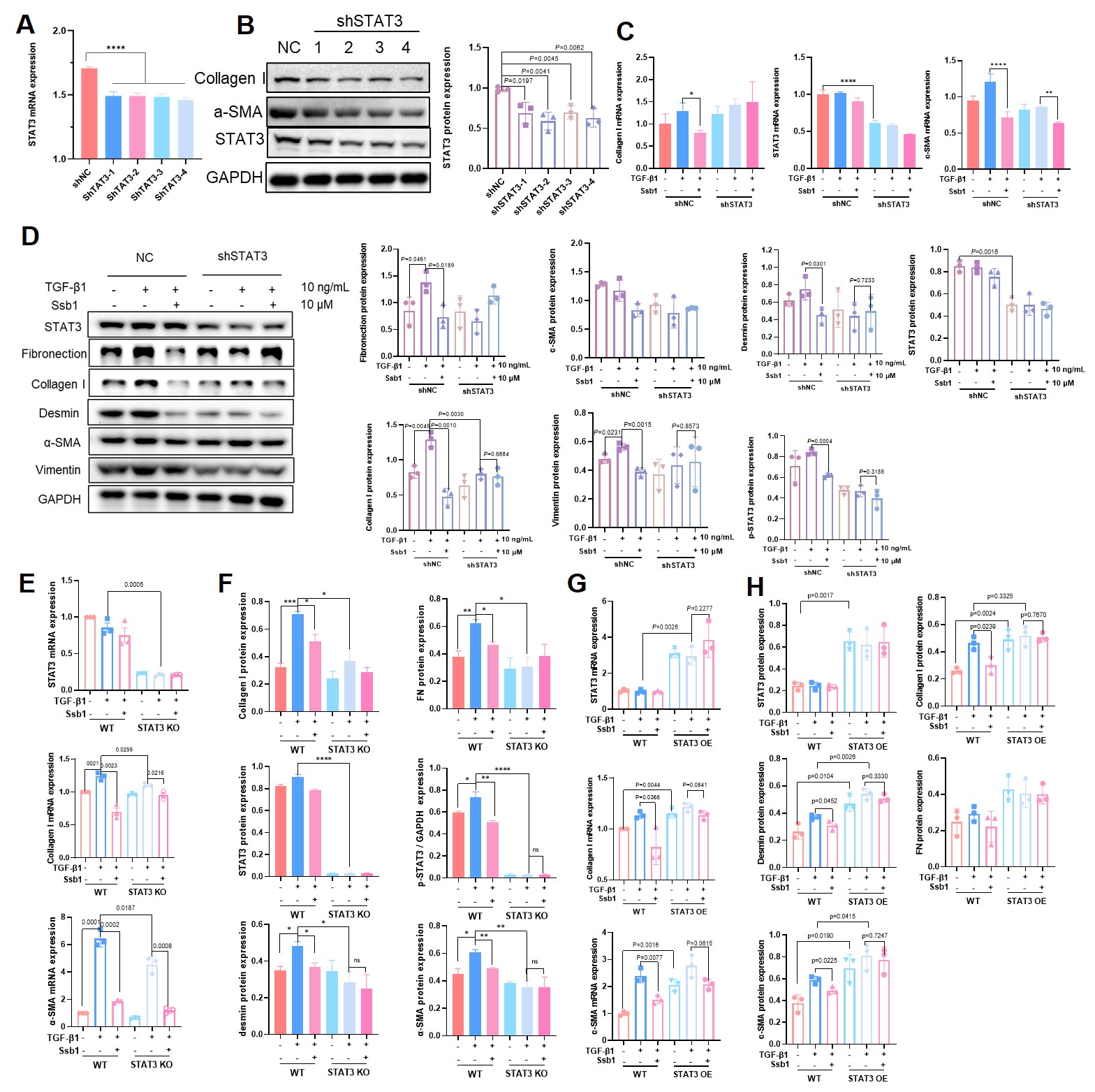


**Figure S8.** HSC-T6 were transfected with shRNA against STAT3. Control cells were transfected with negative control shRNA. (A) RT-qPCR and (B) WB were used to determine knockdown efficiency. (C) mRNA level of Collagen I, α-SMA, and STAT3 following STAT3 knockdown. (D) Protein level of activated HSCs markers following STAT3 knockdown. (E) mRNA level of Collagen I, α-SMA, and STAT3 in STAT3-knockout HSC-T6 treated with Ssb1. (F) Densitometric quantification of protein levels in immunoblots presented in Figure 5H. (G) mRNA level of Collagen I, α-SMA, and STAT3 in STAT3-overexpressed HSC-T6 treated with Ssb1. (H) Densitometric quantification of protein levels in immunoblots presented in Figure 5I.


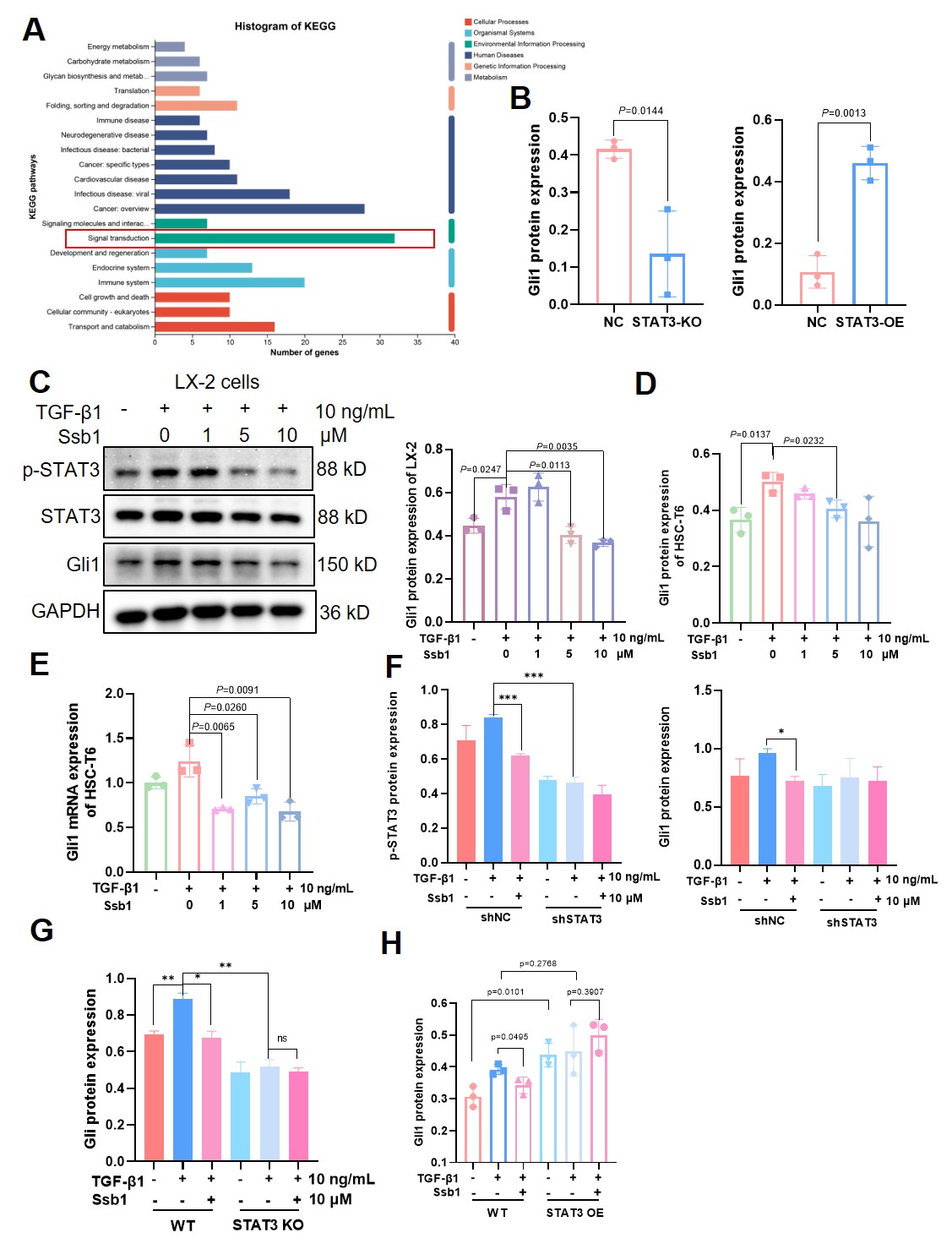


**Figure S9.** (A) Histogram of KEGG enrichment analysis of RNA-sequencing data from differentially expressed genes. (B) Densitometric quantification of protein levels in immunoblots presented in Figs. 6G–H. (C) Protein levels of Gli1 in LX-2 treated with TGF-β1 and different Ssb1 concentrations for 24 h. (D) Densitometric quantification of protein levels in immunoblots presented in Figure 6K. (E) mRNA levels of Gli1 of HSC-T6 treated with TGF-β1 and different Ssb1 concentrations for 24 h. (F–H) Densitometric quantification of protein levels in immunoblots presented in Figs. 6L, 6N, and 6P.


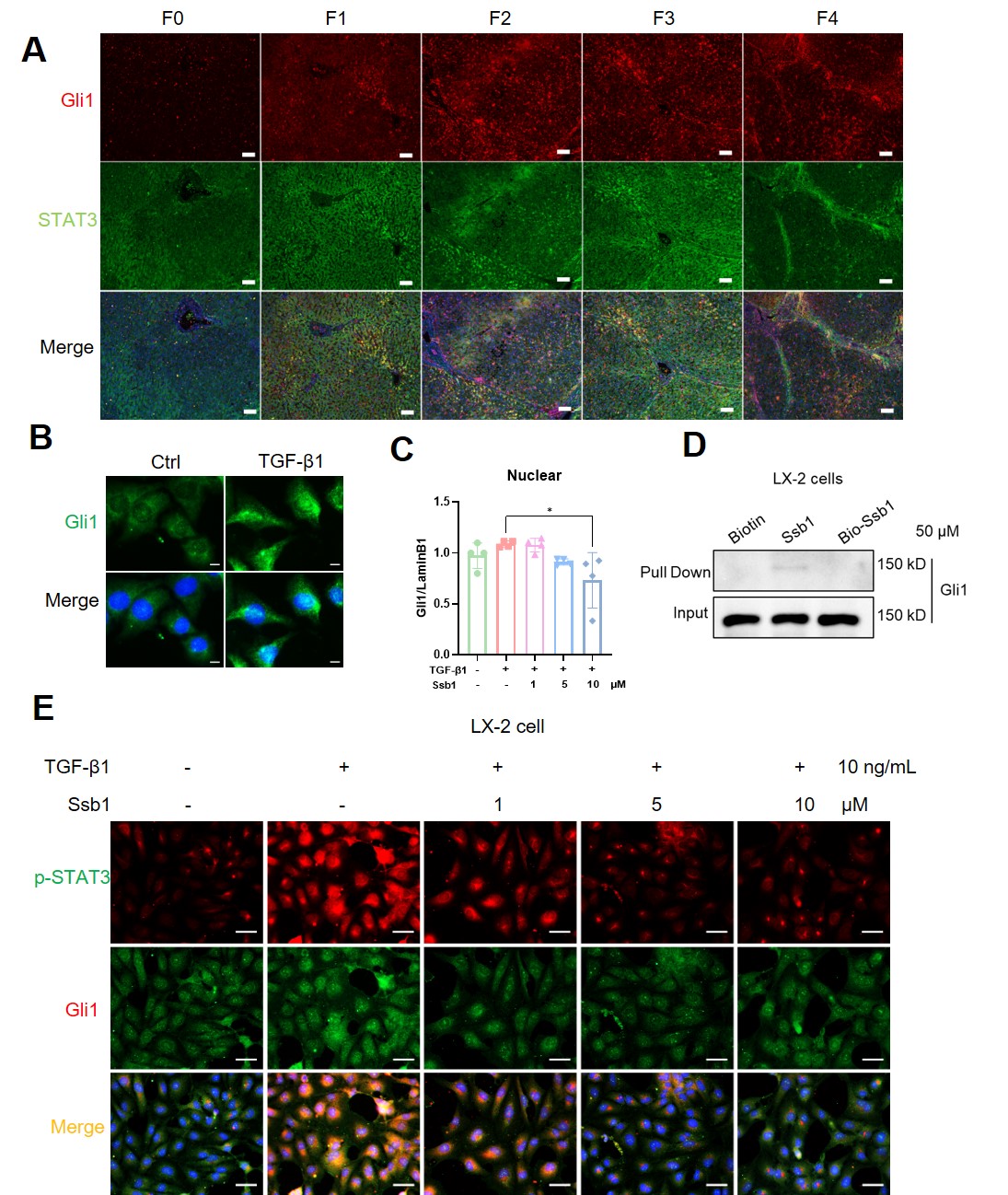


**Figure S10.** (A) Representative dual immunofluorescence of STAT3 and Gli in Clinical human liver tissues with different stage of liver fibrosis. Scale bars, 50 μm. (B) Immunofluorescence analysis of Gli1 distribution in HSC-T6 after TGF-β1 stimulated. Scale bar: 10 μm. (C) Densitometric quantification of protein levels in immunoblots presented in Figure 7F. (D) Pull-down assay of Ssb1 and Gli1 in LX-2. (E) Immunofluorescence analysis of expression and intracellular localization of Gli1 and pSTAT3 in LX-2 with TGF-β1 and Ssb1 treatment. Scale bar: 10 μm.


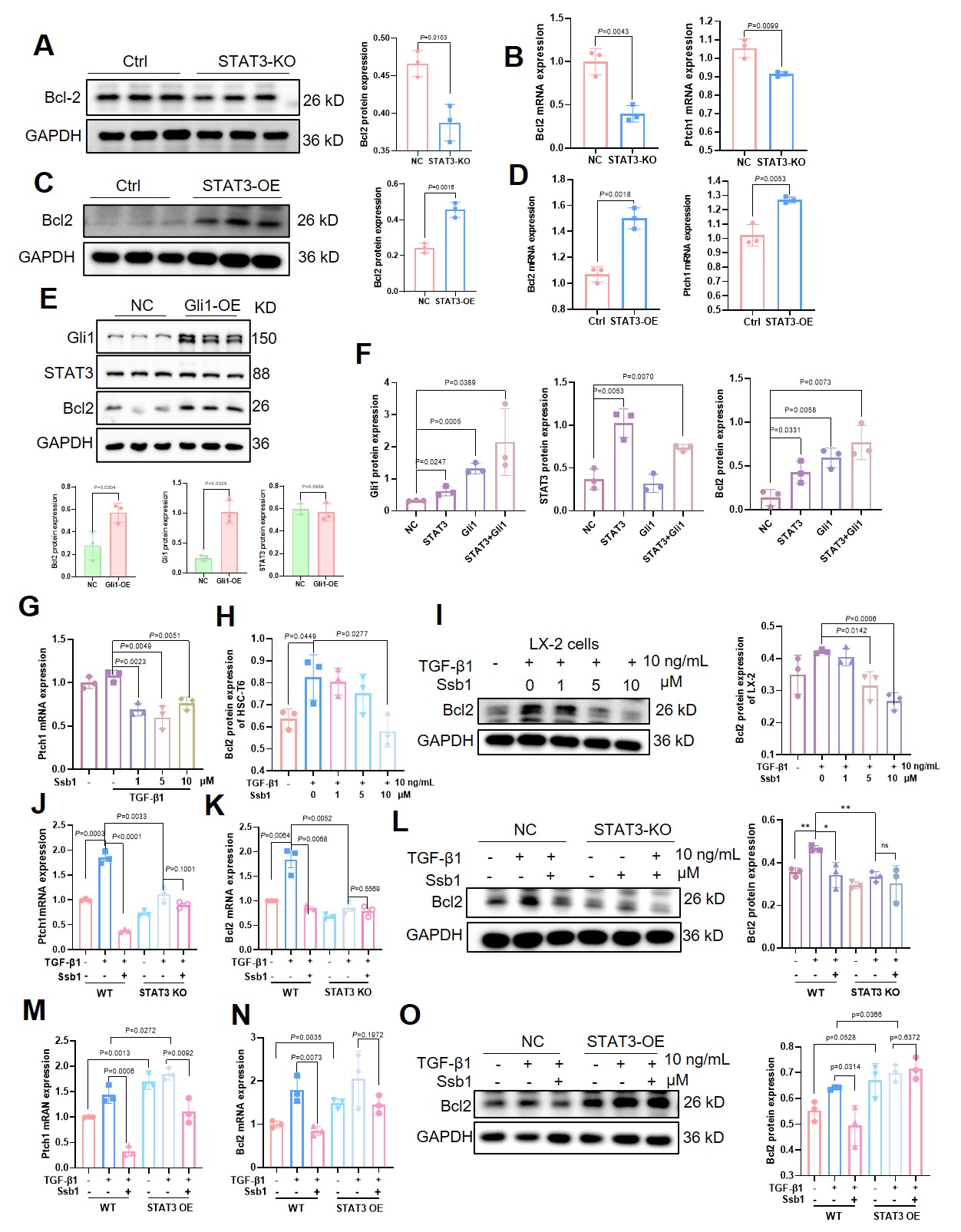


**Figure S11.** (A-D) The mRNA level of Bcl2 and Ptch1 following STAT3 knockout or STAT3 overexpression. (E) The expression of STAT3 and Bcl2 following Gli1 overexpression. (F) Densitometric quantification of protein levels in immunoblots presented in Figure 8B. (G) The mRNA level of Ptch1 after treatment of HSC-T6 cells with different Ssb1 concentrations. (H) Densitometric quantification of protein levels in immunoblots presented in Figure 8D. (I) The protein levels of Bcl2 after treatment of LX-2 cells with different Ssb1 concentrations. (J) The mRNA levels of Ptch1 following STAT3 knockout. (K-L) The mRNA (K) and protein (L) levels of Bcl2 following treated with TGF-β1 and Ssb1 for 24 h in STAT3 knockout HSC-T6. (M) The mRNA levels of Ptch1 following STAT3 knockout. (N-O) The mRNA level of Ptch1 following treated with TGF-β1 and Ssb1 for 24 h in STAT3 overexpression HSC-T6.


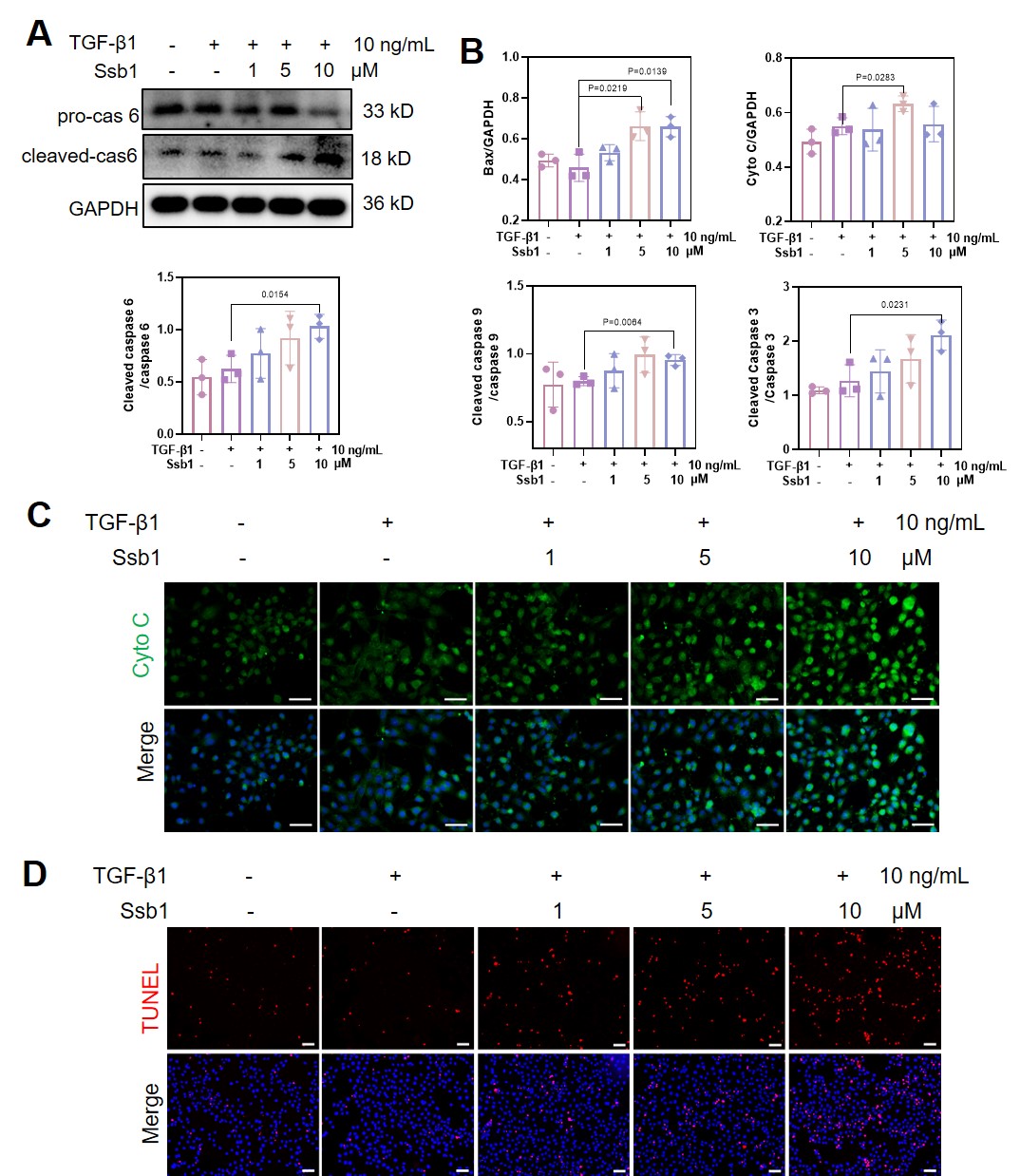


**Figure S12.** (A) The protein expression levels of CytoC, Bax, cleaved‐Caspase‐6. (B) Densitometric quantification of protein levels in immunoblots presented in Figure 8H. (C) Immunofluorescence analysis of CytoC expression in HSC-T6 with TGF-β1 and Ssb1 treatment. Scale bar: 10 μm. (D) TUNEL assay was conducted to observe cell death. Scale bar: 10 μm.


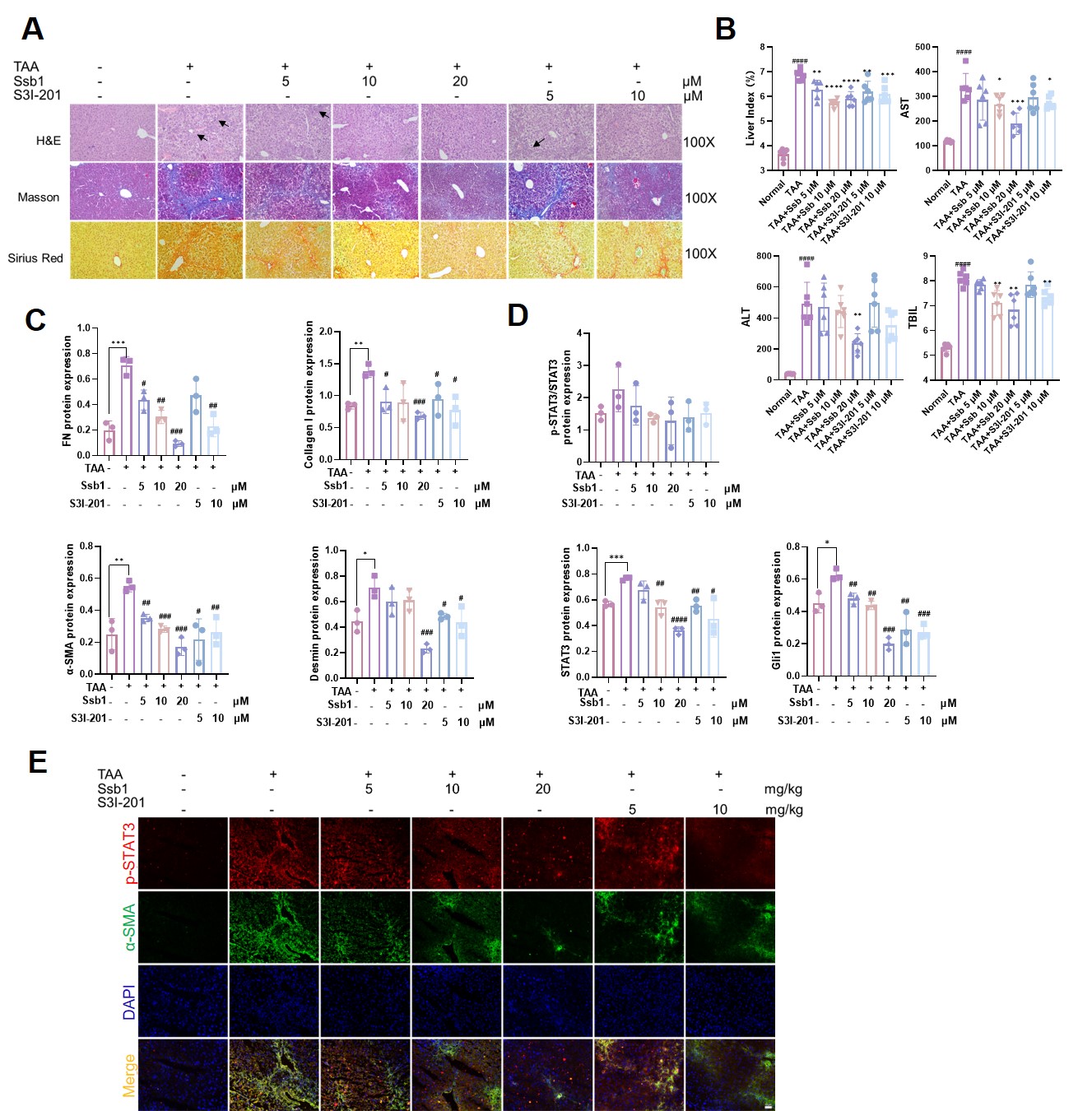


**Figure S13.** Ssb1 ameliorates liver fibrosis in TAA-induced mice. (A) Representative liver cross-section stained with HE, scale bar, 50 μm, Masson’s trichrome scale bar, 50 μm, and Sirius Red, scale bar, 50 μm. (B) Liver index statistics of mice in each group and concentrations of ALT, AST, and TBIL in mouse serum. Each group contained at least six mice. (C) Densitometric quantification of protein levels in immunoblots presented in Figure 9A. (D) Densitometric quantification of protein levels in immunoblots presented in Figure 9B. (E) Images of immunofluorescence staining with p-STAT3 and α-SMA scale bar, 50 μm.


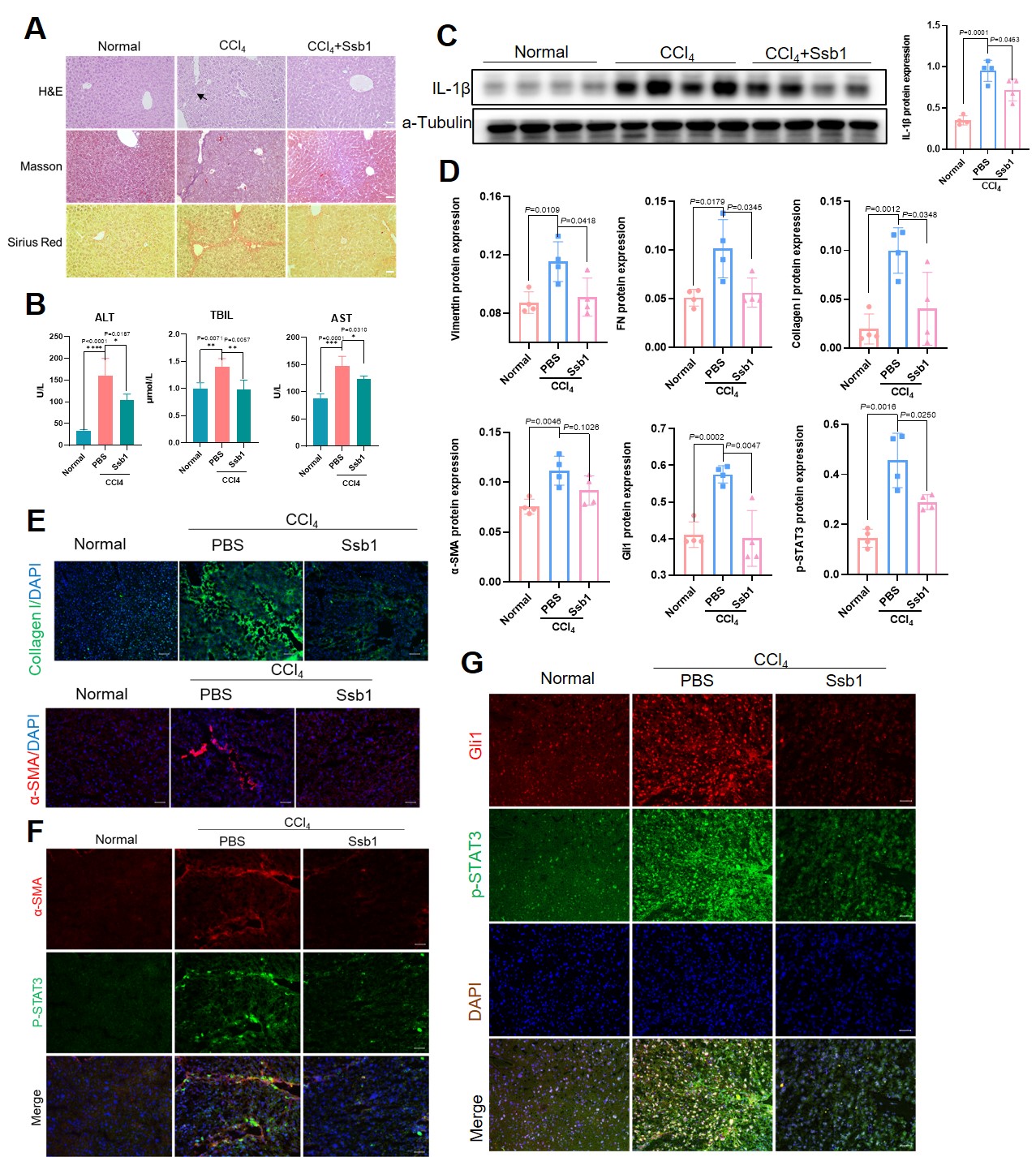


**Figure S14.** Ssb1 ameliorates liver fibrosis in CCl_4_-injected mice. (A) Representative liver cross-section stained with HE, scale bar, 50 μm, Masson’s trichrome scale bar, 50 μm, and Sirius Red, scale bar, 50 μm. (B) The concentrations of ALT, AST, and TBIL in mouse serum. (C) Relative protein levels of IL-1β in mouse livers, as measured by WB. (D) Densitometric quantification of protein levels in immunoblots presented in Figs. 9D-E. (E) Images of immunofluorescence staining of Collagen I and α-SMA. Scale bar, 50 μm. (F–G) Images of immunofluorescence staining with (F) p-STAT3 and α-SMA as well as (G) p-STAT3 and Gli1. Scale bar, 50 μm.


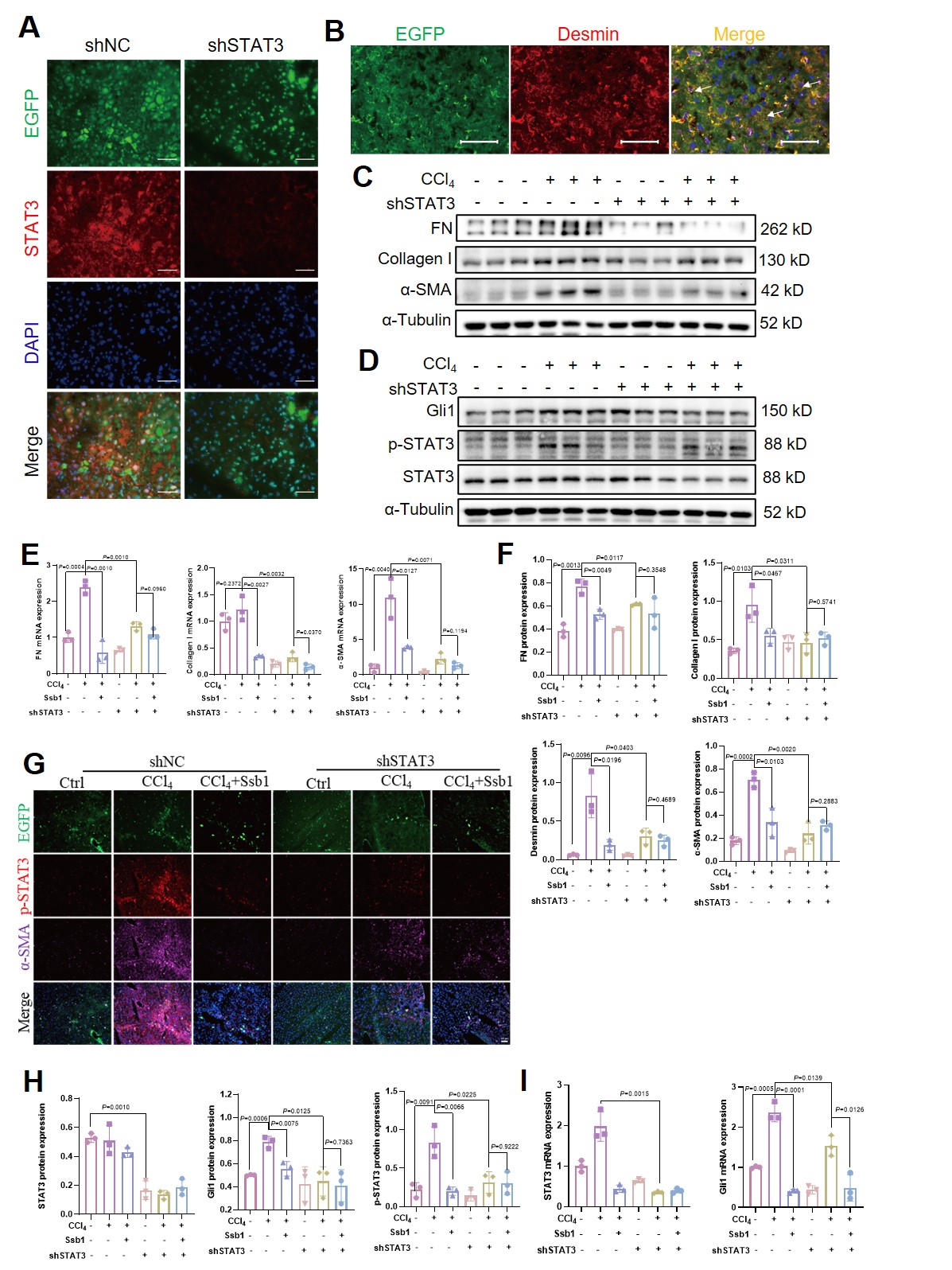


**Figure S15.** (A) Representative fluorescent images of EGFP green and IF for STAT3 red in AAV8-shSTAT3-treated livers. Scale bars, 50 µm. (B) Representative fluorescent images of the co-localization of EGFP green and IF for Desmin red in AAV8-shSTAT3-treated livers. White arrows indicated Desmin^+^ EGFP^+^ cells. Scale bars, 50 µm. (C) Protein levels of FN, Collagen I, Desmin, α-SMA in AAV8-shSTAT3-treated CCl_4_-induced fibrotic livers. (D) Protein levels of P-STAT3, STAT3, and Gli1 in AAV8-shSTAT3-treated CCl_4_-induced fibrotic livers. (E) mRNA levels of the indicated genes FN, Collagen I and α-SMA after Ssb1 treatment in the liver determined by qRT-PCR. (F) Densitometric quantification of protein levels in immunoblots presented in Figure 9J. (G) Immunofluorescence staining with p-STAT3 and α-SMA scale bar, 50 μm. (H) Densitometric quantification of protein levels in immunoblots presented in Figure 9K. (I) mRNA levels of STAT3 and Gli1 in the liver determined by qRT-PCR.

Supplementary Table. List of primers used for qRT-PCR

| Gene names | Primer | Sequence |
| --- | --- | --- |
| Rat β-actin | Forward | 5'-CCCGCGAGTACAACCTTCTTG-3' |
| Rat β-actin | Reversed | 5'-TCATCCATGGCGAACTGGTGG-3' |
| Rat α-SMA | Forward | 5'-GAGGGATCCTGACCCTGAAG-3' |
| Rat α-SMA | Reversed | 5'-CCACGCGAAGCTCGTTATAG-3' |
| Rat COL1A1 | Forward | 5'-TGCCGTGACCTCAAGATGTG-3' |
| Rat COL1A1 | Reversed | 5'-CACAAGCGTGCTGTAGGTGA-3' |
| Rat Vimentin | Forward | 5'- AGATCGATGTGGACGTTTCC -3' |
| Rat Vimentin | Reversed | 5'- CACCTGTCTCCGGTATTCGT -3' |
| Rat HIF-1α | Forward | 5'- AAGTCTAGGGATGCAGCAC -3' |
| Rat HIF-1α | Reversed | 5'- CAAGATCACCAGCATCTAG -3' |
| Rat STAT3 | Forward | 5'-AACGACCTGCAGCAATACCA-3' |
| Rat STAT3 | Reversed | 5'-TCCATGTCAAACGTGAGCGA-3' |
| Rat Gli1 | Forward | 5'-AACTCCACGAGCACACAGG-3' |
| Rat Gli1 | Reversed | 5'-GGCAGTCCGTCTCATACACA-3' |
| Rat Bcl-2 | Forward | 5'-CGGTGGTGGAGGAACTCTTCA-3' |
| Rat Bcl-2 | Reversed | 5'-CGGTTCAGGTACTCAGTCATCCA-3' |
| Rat PTCH1 | Forward | 5'-GGAGCTCAGGCACTATGAAGCAC-3' |
| Rat PTCH1 | Reversed | 5'-TGATGTCTGGAGTCAGGATGCAC-3' |
| Mouse β-actin | Forward | 5'-GACCCAGATCATGTTTGAGAC-3' |
| Mouse β-actin | Reversed | 5'-GTAGCCACGCTCGGTCAG-3' |
| Mouse FN | Forward | 5'-CCACCATTACTGGTCTGGAG-3' |
| Mouse FN | Reversed | 5'-GTCATACCCAGGGTTGGTGA-3' |
| Mouse Bcl2 | Forward | 5'-GGAGAGCGTCAACAGGGAG-3' |
| Mouse Bcl2 | Reversed | 5'-AGACAGCCAGGAGAAATCAA-3' |
| Mouse STAT3 | Forward | 5'-CAACGACCTGCAGCAATACC-3' |
| Mouse STAT3 | Reversed | 5'-CGTGAGCGACTCAAACTGC-3' |
| Mouse COL1A1 | Forward | 5'-CGATGGATTCCCGTTCGAGT-3' |
| Mouse COL1A1 | Reversed | 5'-CGATCTCGTTGGATCCCTGG-3' |
| Mouse α-SMA | Forward | 5'-GTGACTACTGCCGAGCGT-3' |
| Mouse α-SMA | Reversed | 5'-TTTCGTGGATGCCCGCTG-3' |
| Mouse gli1 | Forward | 5'-CCAAGCCAACTTTATGTCAGGG-3' |
| Mouse gli1 | Reversed | 5'-AGCCCGCTTCTTTGTTAATTTGA-3' |
